# Making sense of a pandemic: reasoning about COVID-19 in the intellectual dark web

**DOI:** 10.3389/fsoc.2024.1374042

**Published:** 2024-09-16

**Authors:** Sean Doody

**Affiliations:** National Consortium for the Study of Terrorism and Responses to Terrorism, University of Maryland, College Park, MD, United States

**Keywords:** intellectual dark web, Reddit, social media, collective identity, COVID-19, vaccines, social movements, topic modeling

## Abstract

In this study, I examine how users of an online Reddit community, r/IntellectualDarkWeb, forged an anti-establishment collective identity through practices of “heterodox scientific” reasoning. I do so through a discursive analysis of comments and posts made to r/IntellectualDarkWeb during the COVID-19 pandemic. First, I deploy the BERTopic algorithm to cluster my corpus and surface topics pertaining to COVID-19. Second, I engage in a qualitative content analysis of the relevant clusters to understand how discourses about COVID-19 were mobilized by subreddit users. I show that discussions about COVID-19 were polarized along “contrarian” and “anti-contrarian” lines, with significant implications for the subreddit’s process of collective identity. Overwhelmingly, contrarian content that expressed skepticism towards vaccines, mistrust towards experts, and cynicism about the medical establishment was affirmed by r/IntellectualDarkWeb users. By contrast, anti-contrarian content that sought to counter anti-vaccine rhetoric, defend expertise, or criticize subreddit users for their contrarianism was penalized. A key factor in this dynamic was Reddit’s scoring mechanism, which empowered users to publicly upvote contrarian affirming content while simultaneously downvoting anti-contrarian content. As users participated in sense making about COVID-19, they deployed Reddit’s scoring mechanism to reinforce a contrarian collective identity oriented around a practice of heterodox science. My research shows the continued relevance of the concept of collective identity in the digital age and its utility for understanding contemporary reactionary social movements.

## 1 Introduction

In 2020, the COVID-19 pandemic thrust the world into a global public health crisis. Quickly, it devolved into a political and epistemic one. Governments, experts, and the medical establishment were tasked with not only learning about a novel pandemic virus shrouded in uncertainty (Whooley and Barker, 2021; Pertwee et al., 2022), but also responding to it in real time. Critically, the pandemic emerged in an epistemically unsettled and skeptical moment characterized by what Dean (2019), building on Žižek (2000), calls a “decline in symbolic efficiency”—a breakdown of shared norms, values, and common understandings that make social reality collectively knowable (p. 332). Widely held anti-elite, anti-expert, and anti-establishment sentiments interact precariously with the “superabundance” and “hyper-accessibility” of information in digital networks to cast a “cloud of suspicion” over “all claims to knowledge” (Brubaker, 2017, p. 378). With the weakening of communal structures spearheaded by neoliberalism (Jepperson and Meyer, 2021), self-reliant individuals must now navigate a disorienting epistemic terrain, individually arbitrating disparate and conflicting information in their pursuits of knowledge (Ma, 2024). Lacking trust in legacy epistemic authorities, many now gravitate to alternative sources of authority on digital platforms who are deemed more credible, authentic, and trustworthy (Lewis, 2018, 2020; Finlayson, 2021).

This paper considers the emergence of one such alternative source of authority during the COVID-19 pandemic, the so-called Intellectual Dark Web (IDW). The IDW is a group of heterodox public thinkers loosely held together by a network of podcasts, YouTube channels, and new media publications like *Quillette*. Infamously described by Weiss (2018) in a *New York Times* profile as “iconoclasts” and “academic renegades” who have been “purged” from mainstream institutions due to their controversial opinions, the IDW was positioned as a subversive antagonist to an alleged left-wing cultural and ideological hegemony. Sharing much in common with the broader right-wing reaction against “wokeness,” cancel culture, the LGBTQ+ rights movement, and anti-racist activism (Farrell, 2018; Hamburger, 2018), the IDW tried to distinguish itself through its supposed intellectual seriousness, rationalist epistemology, and ability to transcend ideological and tribalist temptations (Doody, 2020; Parks, 2020). Despite its appeals to rationalism, throughout the course of the COVID-19 pandemic, the IDW became a major source of contrarian information about vaccines, the virus, and medical advice. Popular IDW figures routinely broadcast misinformation about the pandemic—on the alleged dangers of mRNA vaccines, on the efficacy of “miracle drugs” like ivermectin in treating COVID-19, and on the so-called “lab leak” theory of viral origin (Browne and Kavanagh, 2021; Baker and Maddox, 2022; Bharti and Sismondo, 2024)—to massive audiences. For their listeners, they became a trustworthy source of insurgent knowledge that was being illegitimately suppressed by a nebulous “establishment.”

In this study, I seek to understand how individuals who engaged with the IDW during the COVID-19 pandemic reasoned about the virus and the public health response. I do so through a discursive analysis of social media content from r/IntellectualDarkWeb, a user-run Reddit community formed around the IDW brand. I leverage computational text analysis to cluster and surface comments and posts pertaining to COVID-19 on the subreddit. I then engage in a qualitative content analysis of highly upvoted and highly downvoted comments and posts from COVID-19 relevant topical clusters to understand how these discourses manifested in concrete discussions. I argue that through their explorations of the pandemic, expressions of vaccine skepticism, and cynical reasoning about the medical establishment, users of r/IntellectualDarkWeb forged a contrarian collective identity (Polletta and Jasper, 2001). Users mobilized their collective identity on the subreddit to engage in a practice of “heterodox science”—a participatory epistemology that actively contests the epistemic authority of mainstream institutions and experts by appealing to the unorthodox wisdom of “the people” (Mede and Schäfer, 2020; Aupers and de Wildt, 2021). Using the scoring affordances of the Reddit platform, users routinely upvoted skeptical content that questioned the vaccines and contested expertise. By contrast, users who sought to challenge contrarian discoursers on the subreddit were routinely downvoted. Reddit’s scoring mechanisms therefore empowered r/IntellectualDarkWeb users to reinforce their emergent contrarian collective identity.

While past research has examined the ideas, rhetoric, and discourses propagated by IDW figures (Brooks, 2020; Lewis, 2020; Richards and Jones, 2021; Larregue and Lavau, 2024; Matos et al., 2024), very little research has examined how consumers of IDW content engage with the IDW identity themselves. Ribeiro et al. (2020) show that users who interact with IDW content on YouTube go on to radicalize into far-right communities over time, but their study focuses on social network dynamics rather than studying the content produced by users themselves. To the best of my knowledge, my study is one of the first sociological analyses of a major IDW community that centers the discourses, content, and collective identity of the users themselves. My research therefore contributes to the social movements literature on collective identity, which remains dominated by studies of progressive social movements (Fominaya, 2010b; Ackland and O’Neil, 2011; Ray et al., 2017; Stewart and Schultze, 2019; Jackson et al., 2020; Egner, 2022; Gerbaudo, 2022; Nasrin and Fisher, 2022), by demonstrating the utility of the concept for understanding “reactionary” online groups (Futrell and Simi, 2004; Perry and Scrivens, 2016; Rohlinger and Bunnage, 2018; Gaudette et al., 2021).^1^

## 2 Literature review

The social context into which both the IDW and the COVID-19 pandemic emerged is one characterized by a “decline in symbolic efficiency” (Dean, 2010). Epistemic anxiety is pervasive and a generalized mistrust towards expertise prevails. No longer requiring that our information be “mediated” by experts, journalists, and “elite” opinion makers (Han, 2017), we are invited, as free individuals, to decide for ourselves what information to attend to, trust, and believe (Pettman, 2015; Dean, 2016). Digital communications technologies sustain a “marketplace of ideas” overflowing with informational surplus, conflicting evidence, and disparate content from de-differentiated sources (Agger, 2004; Dean, 2005; Finlayson, 2021). Epistemic anxiety arises from the sense that our capacity to falsify is “unlimited:” informational surplus and instantaneous access through digital networks means any single piece of information is always at risk of being displaced (Dean, 2010, p. 103).

Scholars have noted how these informational and epistemic changes have affected individuals’ orientations towards science and medicine. Kata (2012) ties the rise of the anti-vaccine movement to the growth of a postmodern medical paradigm where patients are centered as educated consumers “with access to information diversity” who are “empowered” to question expert advice (Kata, 2012, p. 3779). Patients can easily go online and find information about different procedures and pharmaceuticals that enumerate their risks without mediation through medical experts. For example, publicly accessible preprint servers give anyone access to models, data, and results from new lines of research, generally before they have been peer reviewed (Brubaker, 2021). As Kata (2012, p. 3783) argues, this has led to a “preoccupation of risks over benefits” that encourages individuals to be distrusting and skeptical of medical expertise. To this point, Lasco and Curato (2019) introduce the concept of “medical populism” to describe the growing belief that “ordinary people” are victimized, exploited, and put at risk by the medical establishment. During medical emergencies like the COVID-19 pandemic, this sense of risk provides the normative basis for “the people” to reject novel interventions from medical authorities—such as rapidly developed mRNA vaccines—for unproven treatments that are advertised as simpler and safer (Lasco and Yu, 2022).

Lacking faith in epistemic authorities, “ordinary people” substitute expert opinion for their own “common sense,” life experiences, and “gut feelings” (Mede and Schäfer, 2020). This has encouraged the rise of anti-elite and intuitive epistemics that further erode trust in institutional expertise. For example, Carlson and Ramo (2023, p. 1668) illustrate how conspiracy theorizing among American conservatives during the COVID-19 pandemic functioned as an epistemological practice that cultivated a “self-assured skepticism” that stabilized meaning, provided a sense of control, and offered epistemic relief. Similarly, in their study of COVID-19 conspiracy theories on Twitter, Greve et al. (2022) found that participation in conspiratorial reasoning functions as a “sensemaking” practice for those who distrust “the official story” as told by the government, media, and other establishment institutions. Schradie’s (2019) research on online right-wing social movements shows that conservative activists fluently utilize social media platforms to spread an anti-establishment message that centers their viewpoints and valorizes their skepticism towards mainstream institutions and experts.

This reflects a larger historical pattern of right-wing resentment towards mainstream cultural and political institutions, which have long been seen as ideologically captured by liberal elites who are disdainful of conservative values (Blee and Creasap, 2010; Major, 2015). As Benkler et al. (2018) show, these sentiments are nurtured and spread through a prolific right-wing media ecosystem that links together social media platforms, fake news websites, and conservative media outlets in a sprawling network saturated with ideology-confirming propaganda. Consumption of content from this right-wing media ecosystem is positively linked to beliefs in multiple conspiracy theories, including QAnon, election denialism, and COVID-19 conspiracies (DiMaggio, 2022; Conner, 2023). Motta et al. (2020) found that beginning early in the COVID-19 pandemic, conservative media routinely spread misinformation about the virus and downplayed its harms. In another study, Motta and Stecula (2023) show that conservative media provided more negative coverage of the COVID-19 vaccines than mainstream news, and that this coverage correlated with negative vaccine attitudes among the population.

The implications of these ideational patterns for public health behaviors are significant. Trujillo et al. (2024) present evidence that skepticism towards the COVID-19 vaccines is spilling over into general anti-vaccine attitudes. Similarly, Perlis et al. (2023) found that individuals who endorsed COVID-19 misinformation and expressed distrust towards mainstream medicine were more likely to medicate with unproven COVID-19 treatments like ivermectin or hydroxychloroquine. However, these findings do not indicate simple “echo chamber” effects where unwitting people are mindlessly programmed by the algorithms powering right-wing media ecosystems. Rather, individuals *deliberately* enter media ecosystems to collectively reason with likeminded others about contentious, confusing, and polarizing social issues (Lim, 2017; Greve et al., 2022; Munger and Phillips, 2022). In my view, Lim’s (2020) concept of “algorithmic enclaves” better encapsulates the dynamics at play, especially when paired with insights from the literature on collective identity.

### 2.1 Algorithmic enclaves and collective identity

Algorithmic enclaves are “discursive arenas” where individuals “interact with each other and collectivize based on a perceived shared identity online for defending their beliefs and protecting their resources from both real and perceived threats” (Lim, 2020, p. 194). “Algorithmic” denotes the importance of algorithmic processes within the enclave. But importantly, the formation of an enclave is essentially “voluntary” in nature: “members of a certain enclave have agency and play a role in the formation of their own enclave” (Lim, 2020, p. 194). Individuals seek out digital community intentionally. Algorithms indisputably filter and organize content within an enclave, and some algorithms are more opaque than others (*cf.* Zuboff, 2019). But people, too, can consciously utilize algorithms—by posting, tagging, liking, retweeting, voting, and so on—to produce a community identity and pursue collective aims (Mundt et al., 2018; Waisbord, 2018; Gaudette et al., 2021; Greve et al., 2022). In this way, algorithmic affordances are not simply invisible agents of manipulation, but tools used by social and political actors to achieve strategic goals and mobilize cultural and cognitive resources.

Algorithmic enclaves play an important epistemic role as a “mass adaptative response to the decline in symbolic efficiency” (Dean J., 2024). Outside of one’s enclave, reality “is not the same”—words, concepts, and symbols map to entirely different meanings (Dean J., 2024, p. 205). Seeking refuge in an algorithmic enclave is therefore an understandable response “to the absence of shared meaning in digital networks” (Dean J., 2024, p. 205). Gathering in algorithmic enclaves allows groups of individuals to participate in the formation of collective identities that stabilize their symbolic orders and propagate shared meanings *despite* the fragmenting, individualizing, and isolating processes of digital communications.

The concept of collective identity comes from the social movement literature in sociology (Fominaya, 2010a). Collective identity refers to a common sense of “we-ness” that anchors those with “real or imagined shared attributes and experiences” in relation to “one or more actual or imagined sets of ‘others’” (Snow, 2001, p. 3). Implicit in the “we-ness” of collective identity are feelings of “a common cause, threat, or fate” that motivates people to “act in the name of, or for the sake of, the interests of the collectivity” (Snow, 2001, p. 4). The action component of collective identity is central to the concept (Melucci, 2004). As Snow (2001, p. 3) explains, collective identity does not just suggest the “possibility of collective action in pursuit of common interests,” but “invites such action.” By participating in the process of collective identity, individuals signal a specific *social status*: this entails not only announcing their “affiliation” with others, but also making declarations about the “attitudes, commitments, and rules for behavior” that they subscribe to by assuming the identity (Friedman and McAdam, 1992, p. 157).

Some scholars have questioned the continued relevancy of the concept of collective identity in a period of “personalized politics” where individuals, empowered by social networks, are free to participate in collective actions in a flexible manner without ever having to adopt a rigid group or movement identity (McDonald, 2002; Bennett, 2012; Bennett and Segerberg, 2012). However, a voluminous literature illustrates the enduring relevancy of the concept (Gerbaudo and Treré, 2015; Rohlinger and Bunnage, 2018; Gerbaudo, 2022). Despite the individualizing and fragmenting pressures of contemporary society, individuals *do* engage in active attempts to collectivize under a common cause and identity. The use of hashtags to coordinate and elevate otherwise fragmented and isolated conversations (Ray et al., 2017; Jackson et al., 2020; Nasrin and Fisher, 2022), the documenting and sharing of “offline” subversive political acts through digital visual mediums (Khazraee and Novak, 2018; Stewart and Schultze, 2019), the development of memes and coded language (Gerbaudo, 2015; Tuters and Hagen, 2020)—all of these expressions are efforts to forge collective identity and define a symbolic field of action.

As I will illustrate below, on r/IntellectualDarkWeb, users of the subreddit mobilized contrarian discourses about the COVID-19 pandemic to develop a collective identity whose primary mode of collective action was engaging in a practice of “heterodox” scientific reasoning (Aupers and de Wildt, 2021). Users reinforced the emergent collective identity of the subreddit by leveraging Reddit’s scoring affordances. While the upvoting of contrarian content was a core mechanism used by users of r/IntellectualDarkWeb to positively reinforce the subreddit’s collective identity (Gaudette et al., 2021; Helm et al., 2024), equally important was the penalizing of users who challenged contrarian discourses using the downvoting mechanism. Downvoting is a public expression of disapproval that allows for the enforcement of group boundaries, a key component of collective identity (Gamson, 1997; Fominaya, 2010a). Before turning to my methods and results, I briefly introduce the r/IntellectualDarkWeb subreddit and position it in relation to the “official” IDW.

### 2.2 The r/IntellectualDarkWeb subreddit

**Figure 1:**
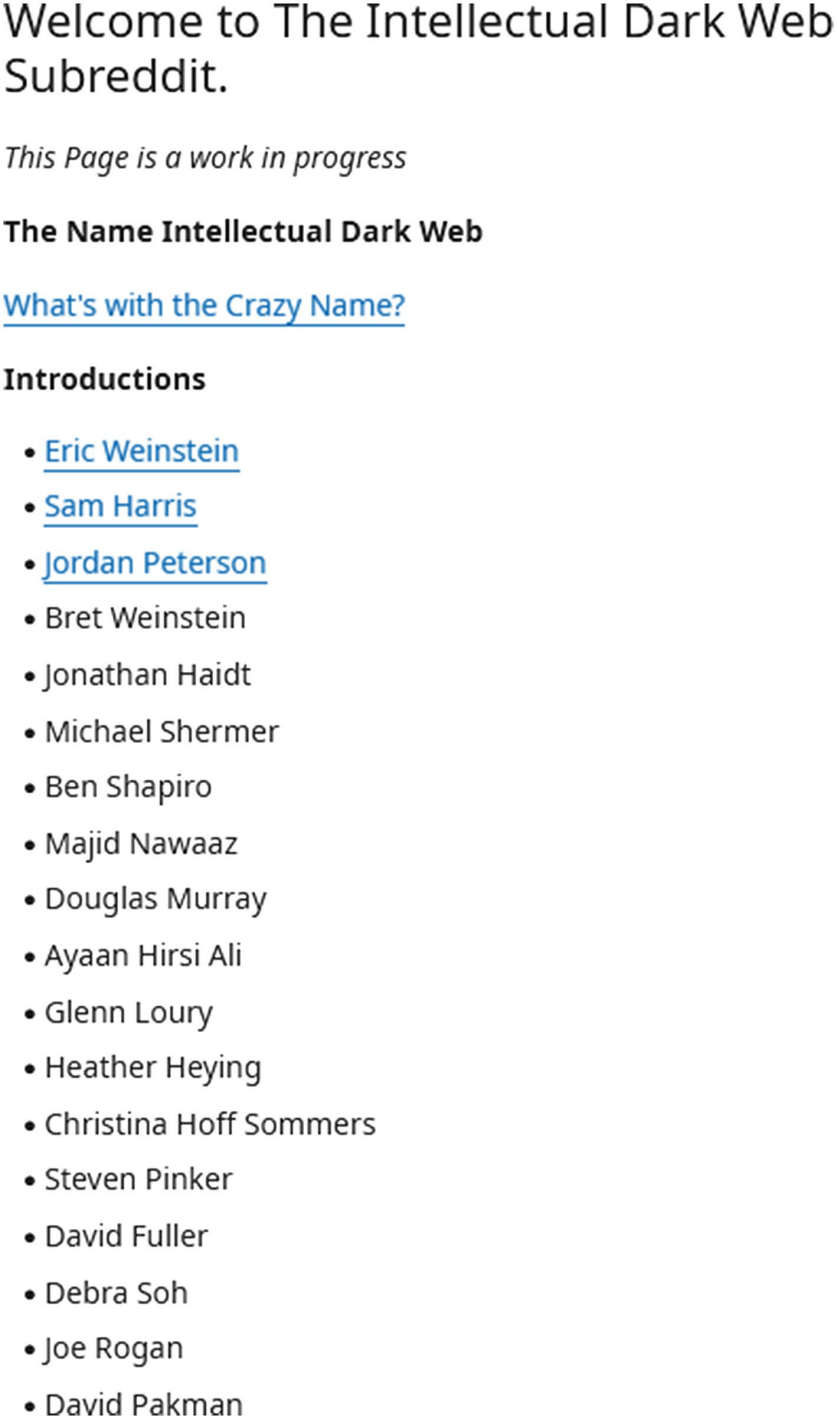
Archived r/IntellectualDarkWeb “Meet the IDW” Page. This screenshot was captured using the Wayback Machine.

r/IntellectualDarkWeb is an online community (“subreddit”) hosted on the Reddit social media platform. According to Statista, in January 2024, Reddit had 5.9 billion worldwide visits (Bianchi, 2024). Recent estimates place the daily number of active Reddit users at 73.1 million, with nearly half being based in the United States (Dean B., 2024). Detailed demographic information about Reddit users is hard to come by, though estimates suggest that it leans young and male (Barthel et al., 2016; Pew Research Center, 2024). The platform has historically had a high tolerance for free speech, including offensive and hate speech, which notoriously allowed for the proliferation of toxic and ideologically extreme communities (Massanari, 2017; Gaudette et al., 2021; Helm et al., 2024). However, in recent years, Reddit has improved its reputation. The platform has banned many of its most toxic communities, implemented a quarantine feature that warns users when they are entering an offensive community, and tightened rules around harassment (Roose, 2024). Controversial subreddits are still allowed to exist, but they face a future where they will likely be required to be attentive to moderating content in their communities.

r/IntellectualDarkWeb came online in January 2018, closely corresponding with the release of a special episode of Sam Harris’s *Making Sense* podcast in which the group was “officially” named by Eric Weinstein (Harris, 2018). During the observation period of this study, r/IntellectualDarkWeb contained a “Meet the IDW” wiki that was meant to introduce subreddit visitors to key figures associated with the group. Although the wiki was never completed and has since disappeared from the subreddit (see the Discussion section below), it features many of the figures named in Weiss’s (2018) *New York Times* profile, as Figure 1 shows.

This list highlights the intellectual diversity of the IDW that ostensibly distinguished it from mainstream conservatism. For example, Eric Weinstein, Bret Weinstein, and Heather Heying are all described by Weiss (2018) as progressives who previously supported Bernie Sanders. The “New Atheist” alum Sam Harris is widely viewed as a center-left liberal, though he finds common ground with the political right over his concerns about Islamic extremism and his support for discriminatory profiling of Muslims (LeDrew, 2016; Brooks, 2020). Popular conservative commentators also appear on the list, including British writer Douglas Murray and the right-wing American pundit Ben Shapiro. Former and current academics critical of social justice activism, such as Jordan Peterson and Steven Pinker, are also named. Peterson is famed for his argument that “postmodern neo-Marxists” have infiltrated academic and cultural institutions and now pose an “existential threat to Western civilization” (Feldmann, 2020, p. 159). While Peterson’s postmodern neo-Marxism has echoes of the “cultural Marxism” conspiracy theory popular with the far-right (Woods, 2019), Peterson’s theory is not explicitly antisemitic. Pinker is a Harvard professor who, like Peterson, is similarly critical of postmodernism’s alleged assaults on Enlightenment rationality (Pinker, 2018; Wesołowski, 2020). Conspicuously absent from the list is Dave Rubin, a conservative YouTube broadcaster who was one of the most enthusiastic supporters of the IDW brand (The Rubin Report, 2018). Rubin fell out of favor with the IDW due to his perceived lack of intellectual rigor (Young, 2019; Brooks, 2020), and this appears to have played a role in why he was not prominently featured on r/IntellectualDarkWeb (Doody, 2022).

The thread that was supposed to hold this disparate band of thinkers together was their joint commitments to classical liberal values, their willingness to engage in civilized debate about any topic, and their commitment to allow truth to reveal itself through rational discussions that transcend tribal boundaries (Weiss, 2018). While this primarily manifested as a unified opposition to the perceived excesses of the academic and activist political left (Wesołowski, 2020; Johansen, 2021), members of the IDW largely viewed themselves as non-tribal proponents of Enlightenment values who were seeking to expand intellectual freedom and challenge mainstream orthodoxies (Shermer et al., 2019; Kelsey, 2020). Sustaining the movement in practice proved to be a heavy burden, however. Since its debut in 2018, the IDW has largely imploded, and many of those affiliated with the brand have openly joined the ranks of the “anti-woke” partisan right (Brooks, 2020; Cammaerts, 2022; Postill, 2024). Other developments, like Sam Harris’s public disavowal of the IDW for its descent into conspiracism, further weakened the public image of the group and fractured the original lineup (Harris, 2020; Young, 2024).

Despite the apparent dissolution of the “official” IDW, the r/IntellectualDarkWeb subreddit has persisted. As of this writing, it remains an open and active community, boasting over 120,000 subscribers and sitting in the top 2% of subreddits by size (see Supplementary Figure 1 for more information about the subreddit’s user activity during this study’s observation period). Moreover, the COVID-19 pandemic provided fertile terrain for individual personalities associated with the IDW to promote their unorthodox ideas about vaccines, virology, and alternative medical interventions to large and skeptical audiences. Bret Weinstein’s and Heather Heying’s *DarkHorse* podcast, along with Joe Rogan’s *Joe Rogan Experience*, became key platforms through which contrarian discussions about the pandemic were staged and misinformation was spread (Browne and Kavanagh, 2021; Baker and Maddox, 2022; Wirstchafter, 2023; Bharti and Sismondo, 2024; Yandell, 2024). And as I will show in the analysis that follows below, users of r/IntellectualDarkWeb actively engaged in contrarian discussions and heterodox reasoning about the COVID-19 pandemic, forging a collective identity that paid homage to the IDW’s founding “iconoclastic” mythos.

## 3 Methods and materials

This research is rooted around two key research questions. First, I ask, how did users of the r/IntellectualDarkWeb subreddit discuss contentious topics pertaining to the COVID-19 pandemic? Second, and building on work by Gaudette et al. (2021), I ask, how did users of r/IntellectualDarkWeb use Reddit’s scoring algorithm (i.e., upvoting and downvoting) to forge a contrarian collective identity rooted around a practice of heterodox science? I answer these questions through a mixed-methods analysis of comments and posts from r/IntellectualDarkWeb pertaining to COVID-19. First, I use the BERTopic algorithm to cluster observations to identify comments and posts discussing the COVID-19 pandemic (Grootendorst, 2022). I then perform detailed qualitative analysis of a sample of observations, coding them for the presence of 12 analytic themes. I discuss my data and methods in detail below.

### 3.1 Data

I collected comments and posts from the Pushshift Reddit Database, a widely used public archive of Reddit data (Baumgartner et al., 2020). In the database, *posts* are submissions by users that start a new thread for discussion. *Comments* are user responses to posts. I collected all posts and comments from r/IntellectualDarkWeb available in Pushshift from the subreddit’s creation in January 2018 through December 31, 2022. As I explain below, while I used the full data to train the topic model, in my content analysis of COVID-19 topics, I focus on observations from January 2020 to December 2022. I extracted 630,963 comments and 18,237 posts from Pushshift. Data collection stopped in 2022 because this was the last full year of complete data available in Pushshift. In 2023, Reddit announced an overhaul of its data API (Spez, 2023; Wiggers, 2023). Because of this change, Pushshift is now only available to subreddit moderators while Reddit develops a new API for academic use cases (Pushshift-Support, 2023; Reddit Staff, 2024).

Reddit’s public scoring algorithm is central to my analysis, and all comments and posts have a score that indicates how positively or negatively they were received by users. Scores are a function of the number of “upvotes” (akin to likes) and “downvotes” (akin to dislikes) a comment or post receives from Redditors (Reddit, 2023). High scores indicate positive reception by the subreddit, while the opposite is true for low scores (which can even be negative). I updated the scores of all comments and posts using the Python Reddit API Wrapper (PRAW) package, as the scores in Pushshift are outdated, only reflecting the score at the time the data was ingested.

### 3.2 Clustering texts with BERTopic

I use the BERTopic algorithm to perform topic modeling on my corpus of Reddit data (Grootendorst, 2022). Historically, the Latent Dirichlet Allocation (LDA) algorithm and its Structural Topic Model (STM) derivative have been the most popular topic modeling techniques in sociology (Blei et al., 2003; Roberts et al., 2014; Fligstein et al., 2017; Karell and Freedman, 2019; Wetts, 2020; Nelson, 2021). However, because LDA depends on word co-occurrences to identify probable topics (Blei, 2012; DiMaggio et al., 2013), the algorithm can struggle when applied to domains like social media characterized by short or noisy text (Greve et al., 2022; Qiang et al., 2022). By contrast, BERTopic makes use of pre-trained transformer language models and density-based clustering to perform topic modeling.

Empirically, BERTopic has been shown to perform very well on social media text (Chong and Chen, 2021; Doody, 2022; Egger and Yu, 2022). The algorithm works by assigning texts to a single topic cluster based on their proximity to other semantically similar observations. First, BERTopic creates a high-dimensional vectorized representation of each text called a document embedding. A document embedding is an output from a language model that represents lexical and semantic features of texts numerically in a vector space. BERTopic leverages the Sentence Transformers Python library, a repository of open-source transformer models trained on more than a billion observations (Reimers and Gurevych, 2019), to create document embeddings. In this paper, I use the all-mpnet-base-v2 model, one of the most performative Sentence Transformers models (Reimers, 2022).

By default, the all-mpnet-base-v2 model truncates texts that contain more than 384 tokens, including two special tokens that indicate the start and end of a text sequence. To account for this, I loaded the model’s tokenizer from Hugging Face and obtained token counts for each comment and post. If a comment or post contained more than 382 tokens, I split the text into *N* chunks, where each chunk has a maximum token count of 382. I then prepended and appended the special tokens “<s>” and “</s>,” respectively, arriving at a maximum length of 384 tokens. For documents consisting of multiple chunks, I compute each chunk as its own document embedding. I then create a single embedding for chunked observations by grouping their embeddings and computing the average of the vectors to obtain one, mean, document embedding.

I applied minimal pre-processing to the texts to remove HTML tags, excessive whitespace, and newline indicators prior to embedding. If Pushshift indicated an observation was removed or deleted, it was discarded. I also discarded observations with less than 10 tokens, as they are unlikely to obtain sufficient information for topic modeling. Finally, I removed all observations originating from a single account that spammed the same message dozens of times to the subreddit, as well as two bot accounts used by the subreddit for auto moderation. For the posts only, I constructed the analytic text by concatenating the title of the post with the “selftext,” or body, of the post. In rare cases, the selftext of some posts indicated they were deleted, but they nevertheless still had accessible titles. In these cases, the text of the post was limited to only the title.

After embedding the documents, BERTopic reduces the dimensionality of the embeddings using the UMAP algorithm to facilitate clustering (McInnes et al., 2020). The resulting UMAP embedding is then fed into the HDBSCAN clustering algorithm to find dense clusters of semantically similar documents (Campello et al., 2013; McInnes and Healy, 2017). Finally, to obtain interpretable text representations of each cluster, BERTopic uses a class-based term-frequency inverse-document-frequency (C-TF-IDF) formula to extract the top *N* weighted terms in a cluster. Specifically, the C-TF-IDF weight *W* for a given term *t* in cluster *c* is defined as

$$W_{t,c}=\;||{tf}_{t,c}||\times \log\left(1+\frac{A}{{tf}_{t}}\right)$$

where *tf_t,c_* is the frequency of term *t* in cluster *c*, *A* is the average number of words across all clusters, and *tf_t_* is the frequency of term *t* across all clusters (Grootendorst, 2022). For each topic, I extract the top 20 words with the highest C-TF-IDF weights to produce human readable topic representations.

There are a few important parameters that affect how BERTopic performs. For the UMAP model, I use the default parameters chosen by BERTopic, which results in the reduction of the 768-dimension all-mpnet-base-v2 embeddings to five dimensions. For HDBSCAN, there are two core parameters: (1) minimum cluster size and (2) minimum samples. Minimum cluster size is the minimum number of observations required to form a cluster. This is a floor and not a ceiling, meaning that HDBSCAN can find clusters of variable sizes that are greater than or equal to this value. The minimum samples parameter relates to HDBSCAN’s concept of noise. As a density-based algorithm, HDBSCAN assumes observations that are distant from other observations are outliers and therefore excludes them from cluster assignment. In practice, this can result in many observations being assigned as noise by HDBSCAN (de Groot et al., 2022). This is deliberate, as HDBSCAN focuses on “being right” rather than maximizing the number of data points clustered (McInnes, 2016a). But it also has the undesirable effect of excluding data points that, upon qualitative review, do seem to belong to a cluster found by HDBSCAN. To decrease noise, the minimum samples parameter can be tuned to make the clustering less conservative (McInnes, 2016b). There is no pure mathematical method for determining the optimal parameters for HDBSCAN. I therefore ran 100 trials of different parameter combinations for minimum cluster size and minimum samples with the goal of both minimizing noise and discovering a plausible number of topical clusters. Through this procedure, I settled on a minimum cluster size of 196 and a minimum samples value of 34. The results from all 100 trials are available in Supplementary Table 1.

With these parameters, BERTopic discovered 255 unique topic clusters. Most of the embeddings were assigned to a cluster, but a sizable minority (47.5%) were classified as noise. To further reduce noise, I follow, with slight modifications, the method used by Angelov (2020) for Top2Vec. First, I create “topic vectors” for each topic cluster, which is equal to the average of all the non-noise document embeddings in a cluster. Second, I calculate the cosine similarities of the noise document embeddings to every topic vector and find which topic vector is most similar to each noise document. Cosine similarity ranges from −1 to +1, with higher scores indicating higher semantic similarity. If the most similar topic vector has a cosine similarity greater than or equal to 0.5, then I assign the noise document to that topic vector. My approach is more conservative than the one used by Angelov (2020) for Top2Vec, where documents are assigned to the cluster of whichever topic vector they are most similar to irrespective of the magnitude of that similarity. Lastly, I compute the cosine similarities of all non-noise documents to their topic vector and eliminate clustered documents whose embeddings are very dissimilar to their respective clusters (similarity <0). After performing this de-noising procedure, 83.3% of the document embeddings were clustered. In total, the BERTopic model was fit on 449,839 observations, or 69.3% of the 649,200 data points collected from Pushshift.

### 3.3 Content analysis

After fitting the BERTopic model, I manually evaluated the results and identified 17 topics relevant to the COVID-19 pandemic. I then performed qualitative content analysis of their comments and posts. These 17 COVID-19 related topics are listed in Table 1 along with their top 20 most highly weighted C-TF-IDF terms (the full topic model specification is available in Supplementary Table 2). To maximize the quality of the topic model’s fit and the coherence of the model’s topics, I used the largest sample size possible to train the BERTopic model. Observations are therefore included from across the data’s entire time-period of January 2018 to December 2022. However, because the first confirmed case of COVID-19 did not occur in the United States until January 20, 2020—several weeks after the CDC’s National Center for Immunization and Respiratory Diseases (NCIRD) began its investigation into the virus following the closure of the Wuhan market on January 1, 2020 (CDC, 2023)—the content analysis of COVID-19 discussions on r/IntellectualDarkWeb is restricted to topically clustered comments and posts from January 1, 2020 to December 31, 2022.

**Table 1:** Summary of COVID-19 related topics from BERTopic model.

Topic ID	Top 20 words
1	vaccine, vaccines, covid, vaccinated, vaccination, immunity, effects, virus, infection, unvaccinated, effective, mandates, deaths, ivermectin, safe, pfizer, myocarditis, fda, disease, adverse
6	ivermectin, drug, treatment, doctors, medical, ivm, studies, covid, trials, doctor, medicine, fda, patients, study, clinical, horse, drugs, treatments, placebo, 19
29	masks, mask, wearing, wear, cloth, mandates, n95, surgical, transmission, spread, covid, droplets, masking, particles, effective, virus, cdc, studies, pandemic, study
63	lab, leak, wuhan, virus, sars, cov, research, viruses, function, gain, bats, china, bat, fauci, origin, coronaviruses, coronavirus, virology, hypothesis, wiv
74	virus, flu, covid, deaths, spread, lockdowns, pandemic, disease, immunity, lockdown, infected, measures, rate, hospitals, health, death, herd, infection, 19, die
96	deaths, covid, death, died, rate, numbers, excess, dying, mortality, die, 19, age, per, 000, million, cdc, hospital, vaccinated, unvaccinated, disease
115	mrna, gene, vaccines, protein, therapy, spike, vaccine, dna, cells, proteins, rna, immune, virus, malone, technology, cell, body, vector, nucleus, effects
167	lockdowns, lockdown, sweden, lock, measures, restrictions, deaths, covid, locked, pandemic, masks, mandates, downs, virus, economy, countries, policies, locking, australia, compliance
168	vax, vaxxers, vaxx, vaxxer, antivax, vaccination, antivaxxers, vaccinated, vaush, vaxxed, pro, covid, antivaxxer, mandate, mandates, vaccinations, medical, antivaxx, hoax, unvaccinated
178	delta, variants, variant, vaccine, virus, vaccinated, vaccines, mutations, infection, immunity, antibodies, mutation, immune, unvaccinated, vaccination, spike, resistant, infected, sars, protein
194	delta, variant, vaccinated, variants, unvaccinated, viral, mutations, spread, omicron, strain, virus, mutation, alpha, load, breakthrough, contagious, infectious, infected, loads, viruses
200	vaccinated, vaccination, vax, vaccinations, unvaccinated, vaxxed, covid, mandate, vaccinate, mandates, vaers, vaccinating, testing, status, mandatory, mandated, require, vaxx, unvaxxed, decision
215	covid, 19, polio, pandemic, contagious, covidiot, catching, booster, denier, mild, covidian, unvaccinated, deadly, lockdowns, covidians, psychosis, spread, illogical, tired, dying
224	immunity, natural, immune, antibodies, infection, cells, antibody, covid, herd, memory, vaccine, vaccinated, disease, vaccination, infected, virus, protection, pathogen, sars, vaccines
234	bret, vaccines, ivermectin, vaccine, brett, covid, heather, roulette, ivm, sam, podcast, weinstein, safe, vaccinated, effective, risks, vax, study, kory, russian
236	vaccinated, virus, infected, unvaccinated, infection, covid, spread, viral, vaccination, symptoms, transmission, asymptomatic, load, viruses, pcr, infections, infectious, immunity, disease, sick
242	hospitals, hospital, beds, icu, patients, hospitalizations, covid, nurses, hospitalization, unvaccinated, capacity, vaccinated, overwhelmed, hospitalized, staff, bed, numbers, medical, healthcare, surgeries

The topic words are derived from the C-TF-IDF algorithm described above. The words are displayed in descending order, with the most important topic words shown first.

**Figure 2:**
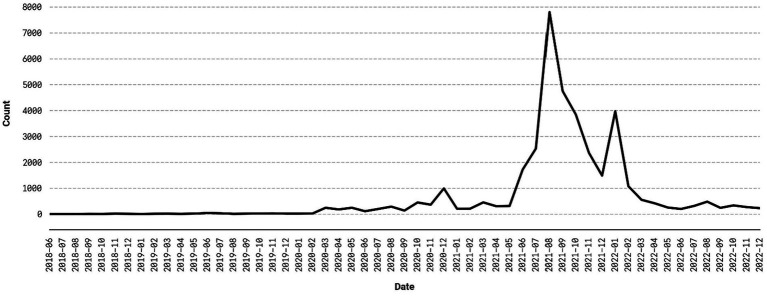
Frequency of COVID-19 topics over time.

Because BERTopic uses semantic similarity to cluster the documents, it is possible that a cluster may contain observations that do not pertain to COVID-19 *specifically* but are otherwise semantically similar to comments and posts in COVID-19 related topics (e.g., a comment discussing vaccines prior to the start of the COVID-19 pandemic). This is not a concern for this study, as the aggregated monthly frequencies of all 17 COVID-19 topics in Figure 2 shows. The frequencies are at or near zero until early 2020—the onset of the pandemic—when they begin to climb. The topics experience a rapid increase in the late spring and early summer of 2021, corresponding with public vaccination campaigns and policies like vaccine checks in public spaces, before declining in frequency in the early months of 2022. Additionally, the top weighted keywords in each of the COVID-19 related topics (Table 1) clearly relate to the pandemic. This suggests that the COVID-19 topics are discriminative, internally coherent, and correspond to the trajectory of the pandemic.

To qualitatively analyze the COVID-19 topic clusters, I sampled 1,000 observations from the aggregated set of comments and posts from the topics in Table 1. Following other researchers, I constructed a “highly upvoted” set of observations consisting of the top 400 most highly upvoted comments and posts from the aggregated COVID-19 topic sample (Gaudette et al., 2021; Helm et al., 2024). Unlike these other approaches, however, I also constructed a “highly downvoted” set of comments and posts, sampling the bottom 400 lowest scored observations from the aggregated set of COVID-19 topics. By sampling the most negatively scored observations in these topics, it becomes possible to engage in a contrastive analysis of which types of discourses are rewarded on the subreddit and which are penalized. Finally, I took a random sample of an additional 200 comments and posts from the aggregate set of COVID-19 topics. To ensure my sample consisted of observations that were most representative of the included topics, all comments and posts sampled for qualitative analysis were required to have a cosine similarity to their topic vector of at least 0.5.

I coded each observation for the presence of 12 discursive categories pertaining to the COVID-19 pandemic, which are defined in Table 2. These codes are not mutually exclusive, meaning multiple categories can be present in a single observation. My approach to coding was similar to the “flexible coding” method described by Deterding and Waters (2021). My method was not “completely inductive” (Deterding and Waters, 2021, p.720), but built on my past research on the IDW (Doody, 2022), cues from my BERTopic model, and the scholarly literature documenting the contentious issues surrounding COVID-19 and the public health response (e.g., Lasco, 2020; Motta et al., 2020; Casarões and Magalhães, 2021; Baker and Maddox, 2022; Motta and Stecula, 2023; Lasco et al., 2024). I began with a preliminary set of analytical codes to guide my initial analysis. I refined, created, and merged categories through multiple readings of the comments and posts, and reviewed entire Reddit threads as necessary to gather additional context. The data was coded using the open source *doccano* annotation tool with subsequent analysis and fine-tuning of the categories occurring in Microsoft Excel.

**Table 2:** Summary of qualitative code categories.

Category	Definition
Alternative Medical Treatments	Comments and posts pertaining to alternative medical treatments for COVID-19, often in lieu of vaccination, which are not recommended by public health authorities (e.g., ivermectin, hydroxychloroquine, vitamin supplementation)
Censorship and Information Suppression	Comments and posts relating to the alleged suppression of information pertaining to the COVID-19 pandemic by different actors (e.g., governments, social media platforms, the medical establishment)
Dangers and Severity of COVID-19	Comments and posts where users discuss how dangerous COVID-19 is to public health, comparisons of COVID-19 to other illnesses, its severity for different populations (e.g., children, young adults, the elderly), and risks from the virus being endemic and mutating
Health Freedom	Comments and posts pertaining to freedoms about individual healthcare decisions and social obligations to public health
Lab Leak, Gain of Function and COVID-19 Origins	Comments and posts pertaining to the origin of COVID-19, including the lab leak theory and discussions of gain of function research
Natural Immunity	Comments and posts that include discussions about the efficacy of natural immunity in preventing future COVID-19 infection, often in contrast to the insufficient immunity of the vaccines
Politicization and Issue Creep	Comments and posts that use COVID-19 discourses as a jumping off point for discussing other politicized, polarizing, or contentious issues
Public Health Policies	Comments and posts pertaining to public health policies, including lockdowns, masking requirements, vaccine passports, and employer and governmental vaccine mandates
Risks and Dangers of the Unvaccinated	Comments and posts discussing the risks that unvaccinated people pose to public health
The IDW	Comments and posts pertaining to IDW members or explicitly directed at the r/IntellectualDarkWeb subreddit
Trust in Institutions and Experts	Comments and posts that include statements about the trustworthiness of public health institutions, scientists, medical experts, and the pharmaceutical industry
Vaccine Safety, Efficacy and Hesitancy	Comments and posts discussing the safety and effectiveness of the COVID-19 vaccines, as well as expressing skepticism or hesitancy about the vaccines

During my analysis, it became apparent that there was significant polarization and conflict between users in r/IntellectualDarkWeb who were taking “contrarian” positions on COVID-19 issues by contesting or discrediting expert and public health advice (e.g., questioning the safety and efficacy of vaccines, bemoaning the alleged suppression of ivermectin by the medical establishment and social media companies), and those who were adopting decidedly “anti-contrarian” positions that directly challenged the circulation of contrarian rhetoric on the subreddit (e.g., critiquing subreddit users for promoting anti-vaccine beliefs, lamenting how leading IDW figures were boosting medical misinformation). I therefore created mutually exclusive codes for “contrarianism” and “anti-contrarianism” to capture this important dynamic among users. While an observation can have multiple qualitative codes attached to it, it can only ever be anti-contrarian, contrarian, or neutral. For the purposes of this study, anti-contrarianism and contrarianism are not the same as “truthful” and “untruthful,” even if misinformation (or “correct” information) is caught in the mix. Rather, these categories are fundamentally about the implied course of epistemic practice communicated by the posts and comments, or the degree to which they encourage adopting, or resist adopting, anti-establishment and heterodox stances regarding COVID-19 (*cf.* Lasco, 2020; Mede and Schäfer, 2020; Aupers and de Wildt, 2021).

## 4 Results

In the following sections, I present the results from my analysis. I begin by reporting the descriptive statistics about my sample, the distribution of code categories, and the prevalence of contrarian and anti-contrarian comments and posts in the qualitatively analyzed sample. My findings show that upvoted content was overwhelmingly contrarian while downvoted content was overwhelmingly anti-contrarian. Users who challenged contrarian rhetoric that circulated widely on the subreddit were publicly penalized for doing so. Across both contrarian and anti-contrarian observations, discussions of vaccine safety, efficacy, and hesitancy featured most prominently, though this theme frequently co-occurred with other code categories. Through a detailed look at representative examples of comments and posts anchored around subreddit discussions about vaccines, I explore these intersections and illustrate how a contrarian collective identity rooted around a practice of heterodox scientific reasoning emerged on r/IntellectualDarkWeb.

### 4.1 Descriptive statistics

Table 3 presents descriptive statistics about the top 400 most upvoted, bottom 400 most downvoted, and 200 randomly sampled observations constituting the qualitative sample. Within the most upvoted group, 68.5% of observations are coded as expressing contrarianism and 18.5% are coded as neutral. Only 13.0% of the comments and posts in the most upvoted sample are coded as expressing anti-contrarianism. By contrast, the most downvoted sample is overwhelmingly constituted by observations expressing anti-contrarianism (82.2%). While 11.5% of observations in the most downvoted sample are coded as expressing contrarianism, this is 57 percentage points less than in the most upvoted sample. The random sample is more evenly distributed, though a plurality of observations express contrarianism (43.5%) followed by anti-contrarianism (30.0%) and neutral sentiment (26.5%).

**Table 3:** Frequency of anti-contrarianism, contrarianism, and neutral codes across samples.

Category				
Sample Source	Anti-Contrarianism	Contrarianism	Neutral	Total
Most Upvoted	52 (13.0%)	274 (68.5%)	74 (18.5%)	400
Most Downvoted	329 (82.2%)	46 (11.5%)	25 (6.2%)	400
Random	60 (30.0%)	87 (43.5%)	53 (26.5%)	200

**Figure 3:**
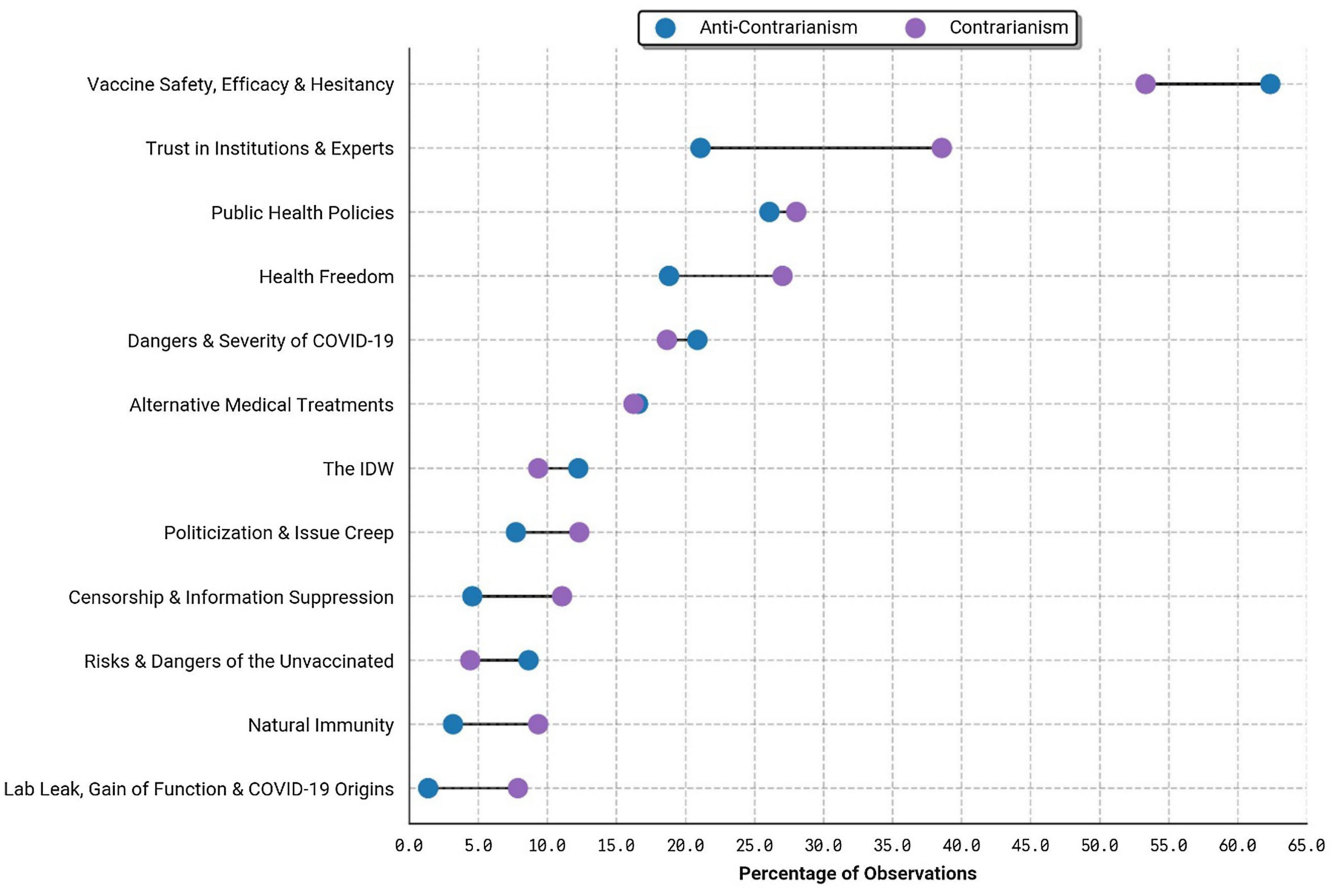
Frequency of code categories in the anti-contrarian and contrarian samples.

Figure 3 plots the frequencies of the 12 code categories across contrarian and anti-contrarian observations. The blue circle is the percentage of anti-contrarian observations that contain the category on the y-axis, and the purple circle is the percentage of contrarian observations that contain the category. By far the most frequent category across both samples is Vaccine Safety, Efficacy & Hesitancy, present in 53.3% of contrarian and 62.4% of anti-contrarian comments and posts. Contrarian content was more likely to be coded for Trust in Institutions & Experts (38.6%) compared to anti-contrarian content (21.1%). A similar share of contrarian and anti-contrarian observations were coded for Public Health Policies (28 and 26.1%, respectively), Dangers and Severity of COVID-19 (18.7 and 20.9%), Alternative Medical Treatments (16.2 and 16.6%), and mentions of the IDW (9.3 and 12.2%). Contrarian observations were more likely to mention Health Freedom (27%) than anti-contrarian observations (18.8%), and they were also more likely to be coded for Politicization & Issue Creep (12.3%) than anti-contrarian content (7.7%). Mentions of Censorship & Information Suppression were more than twice as common in contrarian (11.1%) than anti-contrarian (4.5%) content and mentions of Natural Immunity was nearly three times more common in contrarian (9.3%) than anti-contrarian (3.2%) content. While overall only accounting for a small share of content, the Lab Leak, Gain of Function & COVID-19 Origins category was present in 7.9% of contrarian but only 1.4% of anti-contrarian content. By contrast, anti-contrarian content was nearly twice as likely to mention Risks & Dangers of the Unvaccinated (8.6%) than contrarian content (4.4%).

In the following section, I take a closer look at how these categories manifested in concrete ways in subreddit discussions. Given the domineering prevalence of the Vaccine, Safety & Efficacy category, I use this code as a gateway into examining the contours of COVID-19 discussions on the subreddit. The focus on vaccines is warranted, not only due to the category’s prevalence in my data, but also due to how central anti-vaccine rhetoric has become to some of the most popular IDW figures (Bharti and Sismondo, 2024; Young, 2024). To flesh out how discussion of vaccines fits into the broader discourses pertaining to COVID-19, I focus my analyses on examples highlighting the intersections of the Vaccine Safety, Efficacy & Hesitancy category with the other themes I coded for. Overall, a pattern of upvoting contrarianism and downvoting dissent will be made apparent. In each of the excerpts I illustrate, I provide an anonymized observation ID and the corresponding score. Positive scores are preceded by the “+” symbol, while negative scores are preceded by “-.”

### 4.2 Upvoting contrarianism

Discussions of the vaccines frequently co-occurred with content pertaining to trust in institutions and mainstream expertise. For contrarian content, this often manifested in highly upvoted comments and posts that expressed negative vaccine sentiments and skepticism towards public health authorities and scientists. In many cases, this was paired with a belief that experts and institutions had misled the public about the safety and efficacy of the vaccines:

“What is corrosive of public trust is when public institutions lie … Why did they lie about the vaccines being the ‘safest vaccines ever’? Why did they lie about how the vaccines were going to get us herd immunity and stop the transmission of the virus dead in its tracks?” (ID: C364039, Score: +89).

“If the public health authorities would address their dishonesty in the beginning I would be more interested in listening now. I don't think there are dangerous side-effects of the vaccine, but I don't know because I'm sure if there were they would try to hide them” (ID: C358478, Score +129).

Frequently, users expressed skepticism towards vaccines and experts by focusing on the imperfect efficacy of the vaccines at preventing reinfection. Some users speculated that vaccine imperfections were deliberately engineered by the pharmaceutical industry:

“The very narrow and very short efficacy of the COVID-19 shots should be severely concerning to people. It’s not beyond reason to suspect that this is by design of big pharma to create a permanent and perpetual demand for the shots. It’s common knowledge that they have no interest in curing diseases when they could just treat them long term” (ID: C415130, Score: +82).

It was also common for contrarian content to pair skepticism of vaccines and establishment medicine with appeals to natural immunity:

“Natural immunity is superior … anyone should have been able to deduce in 2020 that natural immunity was superior, and that the claim by the global establishment that vaccine immunity was superior was a lie” (ID: P15450, Score: +181).

These sentiments reflect the rhetorical patterns of “medical populism” described by Lasco and Curato (2019), where mainstream medical institutions are seen as unreliable, untrustworthy, and threatening to public health. Rather than see public health authorities as doing their best amidst the uncertainties of a public health emergency (Pertwee et al., 2022), contrarian users expressed skepticism about the motivations of pharmaceutical companies, cynicism towards the medical establishment, and a presumption that public health authorities were lying to them.

In other cases, contrarian users tied their vaccine skepticism to concerns that medical elites were suppressing credible alternative treatments to promote the vaccines. This often occurred in discussions of drugs like ivermectin that users advocated for despite clinical evidence demonstrating its inefficacy (Naggie et al., 2022; NIH, 2023):

“If ivermectin is proven as an effective treatment of COVID-19 then they don’t have the grounds to authorize a trial vaccine, which is only allowed if there are no proven treatments. Pretty simple to understand the motivation behind ruling ivermectin ineffective” (ID: C327389, Score: +75).

“The vaccines couldn’t have been approved if there was an existing therapeutic [i.e., ivermectin]” (ID: C383723, Score: +45).

“Same with HCQ+zinc [HCQ = hydroxychloroquine] … CAN be taken as a prophylactic, but its primary use is combating Cov19 in those already infected with the virus. So, again, EUA [Emergency Use Authorization] for these experimental ‘vaccines’ should heave been denied. That it wasn’t has costed [sic] tens, more likely hundreds of thousands of lives” (ID: C366707, Score: +17).

In these examples, support for alternative medical treatments is bound up with suspicions about the motivations for approving the COVID-19 vaccines. Doubts are cast on the safety of the vaccines, which are described as “trial” and “experimental,” even though they have been closely monitored by government health agencies. These examples also embody what Lasco and Yu (2022, p. 2) describe as “pharmaceutical messianism,” or the desire for a “quick and easy fix” in the form of a safe “wonder drug.” In the context of COVID-19, ivermectin was championed as a “miracle cure” (Baker and Maddox, 2022), promising safety and medical relief in the absence of “definitive cures” for the virus (Lasco and Yu, 2022). Ivermectin also offered the prospect of shielding individuals from having to expose themselves to the dangers of “experimental” vaccines.

Discourses about health freedom also featured prominently in discussions of COVID-19 vaccines and public health policies. In contrarian comments and posts, this frequently manifested as opposition to any form of COVID-19 vaccine mandates, including in the private sector, as well as mechanisms like vaccine passports that only allow individuals to enter public spaces if they show proof of being vaccinated. These sentiments are epitomized by one of the most highly upvoted comments among contrarian observations:

“The moral problem I run into is telling other people what medical decisions they should make for themselves, based on risk calculations they aren’t participating in … Risk injuries to a few to save many? I don’t know … How many people am I willing to kill by outsourcing violence to the state to fulfill my moral imperative of maintaining public health? While this sounds hyperbolic … any mandate from the government comes from the end of a gun barrel. The state is the only legal arbiter of violence … If you mandate a medical procedure, and a person refuses to participate, what is your answer to that person? … If they resist hard enough, death. So, by my calculations, mandating vaccines for COVID-19 means that you are willing to put some members of society to death in order to prevent a disease that has a 98%+ survival rate. This is bad. You wouldn’t kill for the flu vaccine, and you shouldn’t be willing to kill people for refusing the COVID vaccine either” (ID: C360200, Score: +386).

From this user’s libertarian point of view, any government-enforced vaccine mandate would be equivalent to a promise by the state to kill those who refuse to comply. As the user says, this is a disproportionate response to a virus that has a “98% + survival rate.” Indeed, downplaying the danger and severity of the virus was common in contrarian expressions of health freedom. Frequently, this involved arguing that the virus is not dangerous to young and healthy people, that the vaccine poses more risks than the virus, and the use of folk statistical analysis of public and government statistics to support these claims:

“Right now, the overall death rate [link to Johns Hopkins University’s COVID-19 website] in the U.S. for COVID-19 cases is 1.8% per CDC … 95.2% of those deaths are attributed to those 50 years of age or older. Per recent CDC statistics [link to CDC website] for 2020-2021, if you're between the ages of 18 and 29 … the death rate … is .032%…If we took COVID-19 deaths compared to the 18-29 population as a whole, we arrive at a death rate of .0058%.…Another data point --> we have 6,340 reported deaths [link to CDC website citing VAERS data] from the COVID-19 vaccines out of 177 million … That works out to a .0036% death rate … all COVID-19 vaccines have been empirically untested over the long-term simply because not enough time has passed … because all the vaccines were only subject to EUA [Emergency Use Authorization] approval and not the traditional FDA approval process, the vaccines have not undergone the same level of rigorous testing [as] traditional vaccines … In view of the data above and the philosophical arguments following the data, why should an 18-29 year old person with no preexisting conditions get the COVID-19 vaccine?” (ID: P13805, Score: +194).

In this example, the user engages in their own quantitative analysis of publicly available data to argue that the overall risk of COVID-19 is low for young people and that the vaccines themselves appear to them to be more deadly than the virus. They participate directly in their own heterodox scientific analysis (Aupers and de Wildt, 2021), synthesizing disparate information from publicly available sources online (*cf.* Brubaker, 2021). The claim that vaccines are more risky than the virus is justified with a reference to a CDC source (CDC, 2021) that includes data from the Vaccine Adverse Event Reporting System (VAERS). VAERS is an unverified database of adverse vaccine reports maintained by the U.S. Department of Health and Human Services (HHS). While VAERS has legitimate medical and scientific use cases, it is routinely exploited by anti-vaccine activists to spread doubts about the safety of vaccines (Brumfiel, 2021). And as Motta and Stecula (2023) show, there is a correlation between negative media coverage of COVID-19 vaccines and incidences of adverse events reported in VAERS, leading them to argue that the database functions as a proxy for anti-vaccine sentiment among the public.

Overall, an individualized notion of health freedom, paired with a hesitant and skeptical attitude towards vaccines and other public health initiatives, prevailed in contrarian content. These findings are consistent with other sociological research on vaccine hesitancy. As Reich (2014, p. 697) shows in her study of vaccine refusing parents, parents’ decisions to withhold vaccination from their children is fundamentally about personal choice and individual liberty, and they rarely consider how their unvaccinated children “might present risk to others.” At the same time, the unvaccinated can “free ride” off the immunity of the vaccinated (Reich, 2016). In similar ways, users of r/IntellectualDarkWeb reject any social obligation to compel vaccination, appealing variously to their own individual freedoms and the alleged “negligible” dangers of COVID-19. The virus and the vaccines are treated as a matter of individual risk for people to manage on their own.

### 4.3 Downvoting dissent

In contrast to the upvoted examples explored above, users who engaged in anti-contrarian reasoning on the subreddit were frequently sanctioned by the community. Explicit defenses of vaccines were often met with downvotes, as this comment from a user lamenting the anti-vaccine movement’s efforts to “tear down modern medicine” illustrates:

“One side promotes safe vaccines that slow a deadly virus. The other actively tears down modern medicine, spreads a deadly virus, and then demands to be taken seriously” (ID: C405141, Score: -20).

A similar pattern is observed for defenses of mainstream institutions and public health authorities. Responding to a contrarian post accusing public health authorities of continuously “moving the goal posts” with respect to the required vaccination rate for society “to get back to normal,” this user was downvoted for expressing support for medical leaders who have had to navigate the uncertainty surrounding the pandemic:

“The goalposts have always been, and continue to always be, COVID going down. Nobody knows exactly what level of vaccination will make COVID start going down, but that does not mean the goalposts are moving” (ID: C403441, Score: -11).

Defenses of mainstream science, such as rebuttals to contrarian claims that alternative treatments to COVID-19 like ivermectin were being maliciously suppressed by the medical establishment, were also frequently downvoted:

“Science is a process. Throwing out conspiracies about how working drugs are being suppressed because of money is not the scientific process. Nor is claiming that ivermectin is just as effective as a vaccine without the research and evidence to back it up” (ID: C320365, Score: -8).

It was also common for users to be downvoted for critiquing contrarian subreddit users for believing that high-quality scientific discussion could occur on Reddit and without expert mediation:

“This is just all misconceptions. Scientific debate does not occur on Reddit, and further, scientific debate does not happen amongst laymen … People do discuss the side effects of the vaccine … The claim being made is that it’s 100% clear at the moment that the vaccine is safer than covid” (ID: C377505, Score: -8).

“So you think science should be figured out in comment sections? Let’s say you get heart surgery one day. Do you want your doc to have learned from comment sections? Let’s say you take a high blood pressure pill. Do you want your pharmacist [to] counsel you on that drug based on comment sections? This vaccine is just another drug” (ID: C362388, Score: -10).

Downvoted users also defended actions by social media companies to actively take down content that was promoting anti-vaccine and contrarian talking points, viewing these content moderation activities as a responsible form of harm reduction:

“If people think ivermectin is [as] effective as a vaccine as a result of this video, and forgo the vaccine as a result, that sounds like harm to me” (ID: C320376, Score: -9).

“There is no ‘suppression of ivermectin’. We have a vaccine that’s proven effective. YouTube has a valid reason to suppress videos convincing people to avoid the vaccine in favor of an unproven drug” (ID: C327290, Score: -59).

These examples run directly counter to the types of discourses that were upvoted in the contrarian observations examined above. Rather than see users of r/IntellectualDarkWeb as equipped to engage in complicated scientific analysis, anti-contrarian users emphasize the need to protect the “decision-making sovereignty” and “truth-speaking sovereignty” of experts (Mede and Schäfer, 2020). They express a lack of confidence in the epistemic faculties of “laymen” and reject the notion that legitimate scientific analysis can occur amongst subreddit users.

Interestingly, anti-contrarian comments and posts were slightly more likely than contrarian ones to mention individual IDW figures by name or direct comments explicitly at the subreddit itself. This frequently manifested in downvoted comments where users criticized IDW figures for promoting anti-vaccine ideas:

“Every single time Joe [Rogan] has mentioned the vaccine, and he does a lot, it’s always in a bad light. He’s no longer ‘raising questions’” (ID: C361850, Score: -26).

“798,000 people viewing [Bret Weinstein’s] sketchy pseudoscience that is making people not take the vaccine when the Delta variant is knocking on our door. I’m all for Youtube [sic] taking it down in a time like this” (ID: C324416, Score: -29).

“Risk of covid vaccines are extremely low unlike what IDW morons say. This guy [a reference to Dan Wilson, a molecular biologist who debunks anti-vaccine talking points online under the moniker Debunk the Funk] is an actual scientist, not a biology instructor in a liberal arts college [a dig at Bret Weinstein]” (ID: C470332, Score: -6).

In other cases, users were downvoted for explicitly calling out r/IntellectualDarkWeb for devolving into what they perceived to be an anti-vaccine hub:

“Soo … [sic] this is what this sub is now, huh? Covid is nothing more than mass psychosis and /r/IntellectualDarkWeb is the only place where the few sane people left can be awaken together? I guess I’ll let myself out then” (ID: C361369, Score: -17).

“Oh geez … this has become a dedicated anti-vax sub [reddit] again” (ID: C466806, Score: -18).

Sometimes anti-contrarian discourse boiled over into insults, mockery, or attacks on other r/IntellectualDarkWeb users:

“For a place of ‘free thought and discussion’ idw [sic] seems to be scrambling from one no vaccine solution to the next. Clinging to each faint hope past each scientific consensus [reached] … How many free thinkers died today?” (ID: C406578, Score: -8).

“Misrepresenting data as fucking usual on this sub … You’re a fucking moron who believed in Bret’s dumb theories … You’re all fucking ideologues. There isn’t a shred of intelligence here” (ID: C362053, Score: -14).

Across these examples, we observe what might be called “anti-vax backlash” against the IDW and its supporters. Much like the “mask refusal backlash” Scoville et al. (2022) observed in their analysis of politicized discussions about masking on social media, where antipathy is directed against the “antimaskers” who refuse to wear face coverings to control the spread of COVID-19, anti-contrarian users express disappointment and exasperation towards those they see as proliferating anti-vaccine rhetoric. These opinions are rejected by subreddit users through the accumulation of downvotes, which publicly code these sentiments as out of sync with the heterodox collective identity that was emerging in the community.

On matters of health freedom and public health policies, anti-contrarian content offered perspectives that contrasted to what was common in contrarian comments and posts. Frequently, anti-contrarian users were downvoted for challenging hyperbole about vaccine policies. For example, in response to a post claiming that some workers facing employer vaccine mandates were being “forced to relinquish rights” as if it were the “beginning of the holocaust” (ID: P14476, Score: +119), a commenter challenged this rhetoric, stating, “They do have a choice. Get vaccinated, or do not [work for that employer]” (ID: C399868, Score: −22). Overall, anti-contrarian users tended to emphasize the benefits of mass vaccination for protecting the rights of others beyond the self, as these examples show:

“[But] people also deserve to not be exposed to an unreasonable threat. particularly if they are unable to take the vaccine, or are at high risk and need the support of both the vaccine, and those around them doing everything they can to reduce further spread” (ID: C410161, Score: -18).

“Antivax employees [are] being fired in public sectors because it is against the public’s collective interest. People are free to believe in whatever they want as long as it doesn’t affect others. Why is that so hard to accept?” (ID: C431089, Score: -16).

“Public health measures are not totalitarian for chrissake… [sic] It's not all about you. You live amongst other people your actions affect others THE VIRUS IS AIRBORNE” (ID: C465847, Score: -9).

It was common to see anti-contrarian users argue that there is nothing novel or uniquely threatening about vaccine mandates, as they have existed and continue to exist for other vaccines:

“America has had them [i.e., vaccine mandates] ever since they required school kids to get vaccinated. They're not even new.” (ID: C492320, Score: -9).

“We have had vaccine mandates in several countries for decades. That's how diseases like smallpox, measles and polio were eradicated. Mississippi had the most strict vaccine mandates prior to Covid” (ID: C469761, Score: -11).

“… [it’s] absurd what you've posted on this. Requiring booster passports is completely in line with normal travel around the world in regards to requiring vaccinations. Many countries require various vaccines to enter into the country” (ID: C409401, Score: -16).

In these examples, an emphasis on the rights of the many prevails over the individualized notions of health freedom and the rejection of a social obligation to public health that were common in the contrarian sample. Rather than seeing vaccine mandates and other compulsory health obligations as totalitarian incursions on personal liberty, users who challenged contrarian framing about public health policies saw these as reasonable rules with longstanding precedent. When airing these sentiments publicly on r/IntellectualDarkWeb, these users found themselves being downvoted and rejected by the contrarian weight of the subreddit.

## 5 Discussion

Through their discussions of the COVID-19 pandemic, users of r/IntellectualDarkWeb forged a contrarian collective identity that was put into practice through collective sensemaking about vaccines, public health policies, and the motivations of the medical establishment. The scoring functions of Reddit were mobilized to upvote contrarian and skeptical content, and to downvote those who challenged the subreddit’s emergent contrarian orthodoxy. The anti-establishment ethos of the IDW was channeled by users of r/IntellectualDarkWeb as they collectivized on the subreddit, skeptically reasoned about the pandemic and its fallout, and participated in the collective epistemic practice of “heterodox science” (Aupers and de Wildt, 2021).

My study contributes to the interdisciplinary literature on digital communications and social movements, particularly to a growing line of research exploring online “reactionary” social movements (Lewis, 2018, 2020; Ma, 2024). I show that the concept of collective identity remains useful to understanding social movements despite the transformations of the digital age. Against the personalizing, individualizing, and isolating processes of digital communications (Dean, 2005; Bennett, 2012; Bennett and Segerberg, 2012; Pettman, 2015), individuals *do* engage in the process of collective identity. A voluminous sociological literature shows how collective identity operates through mechanisms like Twitter’s (now X) hashtag functionality to organize discourse and mobilize political action around diverse causes (Brown et al., 2017; Jackson et al., 2020; Egner, 2022; Gerbaudo, 2022; Nasrin and Fisher, 2022). Here, I illustrate how this process works for a contrarian community on Reddit, a platform where users gather in bounded communities with particular expectations for user conduct (Davis and Graham, 2021). The public visibility of Reddit’s scoring algorithm plays an important role in enforcing this conduct, empowering users to fortify their community’s collective identity through upvotes and downvotes (Gaudette et al., 2021).

There are a few important limitations to my study. First, I do not consider how content moderation, either by the subreddit’s own moderators or Reddit itself, affected discourse about COVID-19 on r/IntellectualDarkWeb. During the pandemic, Reddit did enforce some COVID-19 content moderation policies. This included the banning of the r/NoNewNormal subreddit, which was a hub of COVID-19 misinformation on the platform (Worstnerd, 2021). At the same time, Reddit administrators publicly expressed support for allowing “debate, dissent, and protest” about the “mainstream consensus” to remain on the platform as a matter of free speech (Spez, 2021). To this day, the r/IntellectualDarkWeb subreddit remains open and active. However, a recent announcement from r/IntellectualDarkWeb moderators about the “current and future status of the subreddit” ambiguously suggests that Reddit administrators have taken an interest in the happenings of the subreddit, and that r/IntellectualDarkWeb moderators will be “complying with them.” Notably, the subreddit has also stripped itself of previous trappings that explicitly tied it to the “original IDW” brand. As of this writing, the “Meet the IDW” wiki is gone, IDW affiliated podcasts have been removed from the sidebar, and r/IntellectualDarkWeb now describes itself generically as “a subreddit dedicated to discussing politics, history, and social issues.” While these changes are important and warrant further analysis, they are beyond the scope of the current study, having occurred outside the observation period made possible by my data.

Second, my study is primarily focused on the role of discourse and Reddit’s scoring mechanism in the process of collective identity formation. I do not consider what overlaps exist, if any, between r/IntellectualDarkWeb and the globally diffuse anti-lockdown and anti-contagion protests that mobilized large crowds of people under a similar contrarian collective identity (Della Porta, 2023; Ozduzen et al., 2024). I also do not explore the larger Reddit ecosystem users of r/IntellectualDarkWeb are a part of. It would be interesting for subsequent research to map the social networks of users who post contrarian and anti-contrarian content to see if these users belong to different ideological networks on Reddit. This could illuminate important patterns in inter-community conflicts or allegiances (Datta and Adar, 2019). Additional analyses of user dynamics could study whether there is evidence of major “regime changes” on the subreddit at specific moments in time—i.e., periods where different cadres of users drive discussions in particular ways based on their beliefs and behaviors (Kleinberg et al., 2020). This may explain why the highly upvoted sample is not universally constituted by contrarian content and why the highly downvoted sample is not entirely composed of anti-contrarian content (see Table 3).

Despite these limitations, my study provides important insights about how a large online community associated with the IDW, r/IntellectualDarkWeb, reasoned about the COVID-19 pandemic and forged a contrarian collective identity. This collective identity was put into action by users through skeptical discussions of vaccines and the medical establishment. It was further reinforced through a scoring mechanism that allowed users to upvote identity affirming content and downvote anti-contrarian content that challenged prevailing narratives on the subreddit. Overall, my study highlights the importance of the participatory nature of “heterodox” scientific practices on the internet that show no sign of slowing down (Aupers and de Wildt, 2021), and which are likely to continue to have significant implications for how people will choose from where to source information in a period dually characterized by informational abundance and profound epistemic skepticism.

## Data Availability

The code for this analysis is available at: https://github.com/sean-doody/idw-article.

## Footnotes

^1^“Reactionary” is used here to capture the *reactive* nature of groups like the IDW: the positioning of themselves in opposition to the “‘woke’ mob, social justice warriors, and the liberal establishment” (Ma, 2024, p. 200).
