# Corrigendum: Making sense of a pandemic: reasoning about COVID-19 in the intellectual dark web

**DOI:** 10.3389/fsoc.2024.1535266

**Published:** 2025-01-07

**Authors:** Sean Doody

**Affiliations:** National Consortium for the Study of Terrorism and Responses to Terrorism, University of Maryland, College Park, MD, United States

**Keywords:** intellectual dark web, Reddit, social media, collective identity, COVID-19, vaccines, social movements, topic modeling

Regretfully, several typographical and formatting errors were discovered in the published manuscript that need to be corrected.

In the published article, the reference for Doody (2022) was incorrectly written as: Doody, S. (2022). Available at: https://www.proquest.com/docview/2711787652/abstract/B50FFDDCDBD4FF7PQ/1.

It should be: Doody, S. (2022). *Mapping Discourse in the Intellectual Dark Web: A Critical Computational Sociology* [dissertation]. Fairfax, VA: George Mason University. Available at: https://www.proquest.com/docview/2711787652/abstract/B50FFDDCDBD4FF7PQ/1.

In the published article, the reference for Harris (2020) was incorrectly written as: Harris, S. (2020). Republic of lies (225). In Waking Up. Available at: https://www.youtube.com/watch?v=lmcdu6B_YUU.

It should be: Harris, S. (2020). Republic of lies (225). In Making Sense. Available at: https://www.youtube.com/watch?v=lmcdu6B_YUU.

In the published article, the reference for Jepperson and Meyer (2021) was incorrectly written as: Jepperson, R. L., and Meyer, J. W. (2021). “Concluding reflections: evolving cultural models in global and National Society” in *Institutional theory: The cultural construction of organizations, states, and identities*. eds. R. L. Jepperson and J. W. Meyer (Cambridge, MA: Cambridge University Press).

It should be: Jepperson, R. L., and Meyer, J. W. (2021). “Concluding reflections: evolving cultural models in global and national society,” in *Institutional theory: The cultural construction of organizations, states, and identities*, eds. R. L. Jepperson and J. W. Meyer (New York: Cambridge University Press), 297–321.

In the published article, the reference for Barthel et al. (2016) was incorrectly written as: Mitchell, M. B., Stocking, Galen, and Holcomb, Jesse (2016) Reddit news users more likely to be male, young and digital in their news preferences. Pew Research Center. Available at: https://www.pewresearch.org/journalism/2016/02/25/reddit-news-users-more-likely-to-be-male-young-and-digital-in-their-news-preferences/.

It should be: Barthel, M., Stocking, G., Holcomb, J., and Mitchell, A. (2016). Reddit news users more likely to be male, young and digital in their news preferences. Pew Research Center. Available at: https://www.pewresearch.org/journalism/2016/02/25/reddit-news-users-more-likely-to-be-male-young-and-digital-in-their-news-preferences/.

In the published article, the reference for Ozduzen et al. (2024) was incorrectly written as: Ozduzen, O., Aslan Ozgul, B., and Ianosev, B. (2023). ‘Institutions of governance are all corrupted': anti-political collective identity of anti-lockdown protesters in digital and physical spaces. *Soc. Mov. Stud*. 1–19, 1–19. doi: 10.1080/14742837.2023.2246920.

It should be: Ozduzen, O., Aslan Ozgul, B., and Ianosev, B. (2024). “Institutions of governance are all corrupted”: anti-political collective identity of anti-lockdown protesters in digital and physical spaces. *Soc. Mov. Stud*. 23, 676–694. doi: 10.1080/14742837.2023.2246920.

In the published article, the reference for Richards and Jones (2021) was incorrectly written as: Richards, I., and Jones, C. (2021). “Quillette, classical liberalism, and the international new right” in *Contemporary far-right thinkers and the future of Liberal democracy*. eds. A. J. McAdams and A. Castrillon (Routledge), 121, London−148.

It should be: Richards, I., and Jones, C. (2021). “Quillette, classical liberalism, and the international new right” in *Contemporary far-right thinkers and the future of Liberal democracy*, eds. A. J. McAdams and A. Castrillon (London: Routledge), 121–148.

In the published article, the reference for Rohlinger and Bunnage (2018) was incorrectly written as: Rohlinger, D. A., and Bunnage, L. A. (2018). Collective identity in the digital age: think and thick identities in MoveOn.org and the tea party movement. *Mobilization* 23, 135–157. doi: 10.17813/1086-671X-23-2-135.

It should be: Rohlinger, D. A., and Bunnage, L. A. (2018). Collective identity in the digital age: thin and thick identities in MoveOn.org and the tea party movement. *Mobilization* 23, 135–157. doi: 10.17813/1086-671X-23-2-135.

In the published article, the reference for Žižek (2000) was incorrectly written as: Žižek, S. (2000). *The ticklish subject. New York, Verso: The absent Centre of political ontology*.

It should be: Žižek, S. (2000). *The Ticklish Subject: The Absent Centre of Political Ontology*. New York: Verso.

In the published article, the reference for Zuboff (2019) was incorrectly written as: Zuboff, S. (2019). *The age of surveillance capitalism. New York, PublicAffairs: The fight for a human future at the new frontier of power*.

It should be: Zuboff, S. (2019). *The Age of Surveillance Capitalism: The Fight for a Human Future at the New Frontier of Power*. New York: PublicAffairs.

In the published article, the wrong citation was used for the first footnote.

A correction has been made to **Introduction**, Paragraph 4, Footnote 1. This sentence previously stated:

““Reactionary” is used here to capture the *reactive* nature of groups like the IDW: the positioning of themselves in opposition to the “‘woke' mob, social justice warriors, and the liberal establishment” (Bianchi, 2024, p. 2).”

The corrected sentence appears below:

““Reactionary” is used here to capture the *reactive* nature of groups like the IDW: the positioning of themselves in opposition to the “‘woke' mob, social justice warriors, and the liberal establishment” (Ma, 2024, p. 200).”

The author apologizes for these errors and states that this does not change the scientific conclusions of the article in any way. The original article has been updated.

