# Supplementary figures and images for: Making sense of a pandemic: reasoning about COVID-19 in the intellectual dark web
Source: Front Sociol. 2024 Sep 16;9:1374042. doi: 10.3389/fsoc.2024.1374042 (PMC11440435; doi:10.3389/fsoc.2024.1374042)

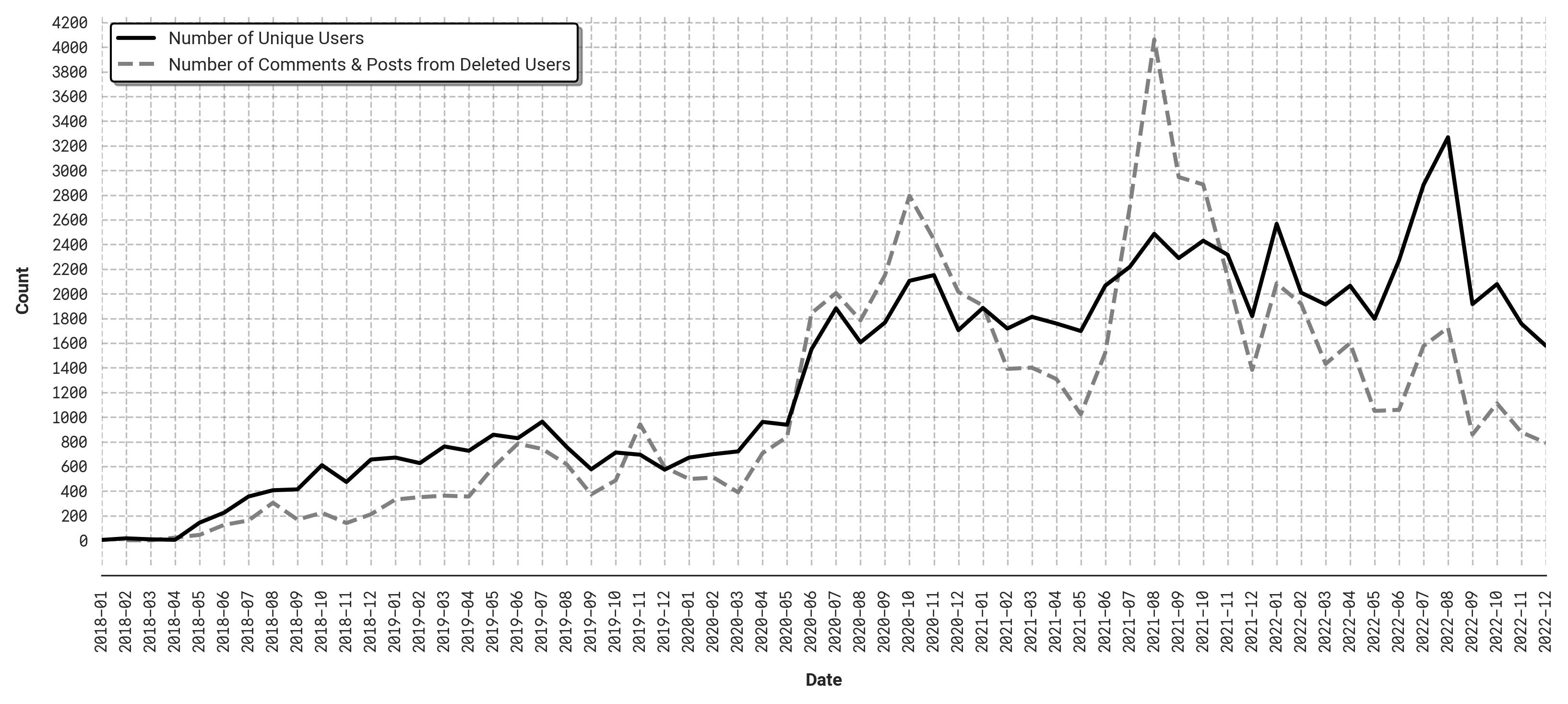

Supplement: Supplementary file 1 [file Image_1.jpg]
