# Supplementary material for: Making sense of a pandemic: reasoning about COVID-19 in the intellectual dark web
Source: Front Sociol. 2024 Sep 16;9:1374042. doi: 10.3389/fsoc.2024.1374042 (PMC11440435; doi:10.3389/fsoc.2024.1374042)
Supplement: Supplementary file 2 [file Table_1.pdf]

Supplementary Table 1: Results of HDBSCAN Clustering Trials

loss	min_cluster_size	min_samples	noise_proportion	k_topics
0.475	196	34	0.475	255
0.482	187	27	0.482	279
0.483	198	48	0.483	249
0.483	185	32	0.483	273
0.484	172	17	0.484	300
0.484	198	30	0.484	262
0.485	196	24	0.485	270
0.485	196	24	0.485	270
0.485	196	24	0.485	270
0.485	180	17	0.485	288
0.486	174	18	0.485	298
0.488	175	32	0.488	287
0.489	188	43	0.489	265
0.490	170	39	0.490	290
0.492	176	25	0.492	298
0.494	192	47	0.494	262
0.495	162	50	0.495	292
0.496	193	23	0.495	275
0.497	186	28	0.496	285
0.497	171	50	0.496	281
0.498	173	50	0.497	278
0.504	187	46	0.504	272
0.505	195	3	0.505	298
0.507	200	10	0.507	275
0.508	190	10	0.508	294
0.522	196	12	0.522	298
0.524	199	5	0.524	289
0.630	123	30	0.479	382
0.630	151	34	0.479	314
0.630	158	34	0.480	305
0.630	157	22	0.480	320
0.635	172	29	0.484	301
0.635	145	27	0.485	340
0.636	143	27	0.485	343
0.637	147	48	0.486	307
0.637	142	37	0.487	329
0.637	133	17	0.487	359
0.637	140	15	0.487	365
0.638	124	38	0.488	369

0.640	141	48	0.489	319
0.641	148	36	0.490	320
0.641	167	20	0.490	304
0.641	144	38	0.490	327
0.642	92	37	0.492	446
0.643	163	13	0.493	326
0.644	68	34	0.493	557
0.644	152	25	0.494	331
0.645	108	36	0.494	411
0.645	127	33	0.494	377
0.646	125	31	0.496	393
0.647	110	39	0.497	405
0.648	60	31	0.497	616
0.648	74	14	0.497	586
0.648	159	16	0.498	320
0.648	85	19	0.498	511
0.649	112	49	0.498	384
0.649	153	40	0.499	317
0.650	156	50	0.500	301
0.650	169	16	0.500	305
0.651	97	29	0.500	455
0.651	149	4	0.501	366
0.652	55	27	0.501	662
0.652	86	48	0.501	448
0.652	166	4	0.502	335
0.652	77	13	0.501	574
0.653	99	21	0.502	469
0.653	101	15	0.502	469
0.654	111	33	0.503	411
0.656	109	21	0.505	447
0.656	137	23	0.506	365
0.656	117	26	0.506	410
0.656	84	21	0.506	527
0.656	138	42	0.506	343
0.657	94	8	0.506	510
0.657	67	40	0.506	556
0.658	135	26	0.507	370
0.658	172	8	0.507	322
0.658	93	41	0.507	453
0.658	70	17	0.508	611
0.659	132	22	0.508	371

0.660	61	34	0.510	610
0.660	116	44	0.510	389
0.661	88	24	0.511	504
0.662	73	46	0.511	515
0.662	164	9	0.512	336
0.662	122	43	0.512	378
0.662	82	35	0.512	499
0.663	120	6	0.513	436
0.663	54	17	0.512	719
0.664	64	2	0.514	836
0.667	100	7	0.516	493
0.668	59	20	0.517	678
0.669	177	12	0.519	319
0.670	51	21	0.519	743
0.672	160	5	0.521	351
0.672	80	11	0.521	591
0.672	172	7	0.522	329
0.673	95	6	0.522	535
0.674	50	41	0.524	678
0.677	53	45	0.527	647
