# Supplementary material for: Making sense of a pandemic: reasoning about COVID-19 in the intellectual dark web
Source: Front Sociol. 2024 Sep 16;9:1374042. doi: 10.3389/fsoc.2024.1374042 (PMC11440435; doi:10.3389/fsoc.2024.1374042)
Supplement: Supplementary file 3 [file Table_2.pdf]

Supplementary Table 2: Full Topic Model Specification

Topic ID	Count	Top 20 Words
0	14871	wing, liberal, conservative, conservatives, progressive, liberals, leftist, progressives, leftists, liberalism, center, party, radical, conservatism, ideology, progressivism, centrist, leaning, progress, moderate
1	12489	vaccine, vaccines, covid, vaccinated, vaccination, immunity, effects, virus, infection, unvaccinated, effective, mandates, deaths, ivermectin, safe, pfizer, myocarditis, fda, disease, adverse
2	10659	gender, trans, sex, woman, transgender, biological, female, dysphoria, male, transition, binary, identity, construct, surgery, men, identify, cis, feminine, biology, hormones
3	11694	podcast, interview, podcasts, episode, guests, listening, listened, interviews, rogan, audience, maher, conservative, talks, guest, topics, videos, youtube, episodes, jbp, smart
4	12210	reply, respond, responding, responded, answered, replied, replying, insult, answering, insults, waste, responses, sentence, wow, engaging, glad, worries, explained, replies, ad
5	8046	religion, religious, christian, christianity, church, religions, atheist, belief, bible, christians, atheists, atheism, supernatural, beliefs, catholic, gods, jesus, secular, universe, existence
6	7944	ivermectin, drug, treatment, doctors, medical, ivm, studies, covid, trials, doctor, medicine, fda, patients, study, clinical, horse, drugs, treatments, placebo, 19
7	7629	twitter, censorship, facebook, platform, platforms, speech, content, companies, users, private, 230, company, tech, banned, musk, parler, ban, elon, censor, online
8	9143	biden, president, obama, bernie, vote, election, candidate, 2016, polls, win, sanders, voting, democrats, voted, clinton, voters, hillary, supporters, party, dnc
9	6360	abortion, fetus, abortions, baby, pregnancy, pro, roe, birth, mother, child, pregnant, autonomy, personhood, body, woman, bodily, unborn, rights, conception, weeks
10	5020	russia, ukraine, putin, russian, nato, ukrainian, invasion, russians, crimea, military, ukrainians, west, sanctions, soviet, propaganda, invade, civilians, western, troops, nukes
11	6415	fox, cnn, journalism, outlets, reporting, journalists, msnbc, mainstream, bias, npr, nyt, biased, outlet, msm, stories, journalist, press, propaganda, coverage, fake

12	6248	riots, protests, capitol, protest, peaceful, riot, rioters, protesters, rioting, violence, insurrection, police, blm, violent, protestors, looting, protesting, portland, 6th, jan
13	9167	racism, racist, systemic, race, racial, whites, color, blacks, racists, skin, discrimination, americans, african, races, minorities, prejudice, outcomes, asians, kendi, supremacy
14	3940	woke, wokeness, wokeism, ideology, wokes, religion, movement, racism, wokism, liberal, justice, racist, progressive, religious, wing, identity, conservatives, cult, cultural, race
15	4449	aoc, tulsi, omar, fired, disney, pelosi, gabbard, greta, israel, twitter, tweet, party, semitic, shes, speech, carano, hillary, interview, tweets, jews
16	3978	climate, carbon, warming, emissions, energy, co2, nuclear, global, fossil, solar, green, scientists, science, fuels, temperature, wind, earth, ipcc, planet, greenhouse
17	4085	peterson, jordan, zizek, marxism, marxist, jp, harris, religion, dr, marxists, lectures, psychology, postmodernism, postmodern, jbp, sam, talks, fans, christian, interview
18	3399	china, chinese, ccp, taiwan, genocide, hong, kong, xinjiang, xi, camps, communist, mao, han, west, trade, western, countries, prc, uighur, uighurs
19	4342	science, scientific, scientists, consensus, experts, scientist, sciences, research, method, field, trust, expert, peer, fields, etica, studies, papers, academia, scientism, sociology
20	4341	police, crime, blacks, cops, crimes, killed, violent, racism, commit, whites, policing, rates, racial, race, statistics, disproportionately, brutality, officers, rate, shootings
21	4610	election, fraud, ballots, votes, mail, voter, ballot, voting, elections, vote, counted, counting, stolen, rigged, voters, results, biden, fraudulent, audit, georgia
22	4793	fbi, investigation, documents, mueller, russian, president, russia, campaign, election, clinton, impeachment, warrant, biden, classified, report, hillary, collusion, assange, investigations, corruption
23	2776	gun, guns, shootings, mass, amendment, weapons, firearms, shooting, firearm, rifles, armed, arms, ownership, 2a, militia, laws, rifle, violence, school, 2nd
24	3941	speech, freedom, amendment, protected, censorship, rights, consequences, expression, violence, laws, fire, legal, private, crowded, censor, hateful, harm, misinformation, 1a, 1st

25	3232	race, genetic, races, differences, genetics, ancestry, populations, skin, traits, genes, biological, racial, construct, color, categories, african, species, genetically, subspecies, variation
26	3808	market, private, companies, corporations, monopolies, markets, regulation, monopoly, company, regulations, gov, profit, sector, competition, monarchy, businesses, governments, corporation, profits, corporate
27	3812	diversity, affirmative, discrimination, hiring, race, applicants, racial, students, quotas, minorities, admissions, training, whites, names, asians, asian, hire, qualified, color, minority
28	2626	iq, intelligence, scores, differences, cognitive, genetic, test, tests, gap, average, race, genetics, murray, measure, racial, intelligent, curve, bell, factors, environmental
29	2348	masks, mask, wearing, wear, cloth, mandates, n95, surgical, transmission, spread, covid, droplets, masking, particles, effective, virus, cdc, studies, pandemic, study
30	3021	university, universities, students, academia, professors, professor, college, campus, faculty, academic, education, student, colleges, humanities, academics, conservative, liberal, school, institutions, stem
31	2162	cancel, cancelled, boycott, canceled, cancelling, cancellation, boycotts, canceling, fired, boycotting, company, mob, dixie, chicks, speech, twitter, goodyear, cancellations, firing, conservatives
32	2335	slavery, slaves, slave, 1619, enslaved, project, chattel, trade, south, colonies, somerset, africans, owners, african, civil, reparations, africa, jefferson, indentured, plantation
33	3830	banned, mods, ban, subs, subreddit, mod, users, banning, block, subreddits, bans, moderators, user, blocked, moderator, posting, brigading, blocking, trolls, content
34	2316	healthcare, insurance, health, medicare, costs, pay, doctors, cost, medical, universal, socialized, private, payer, market, doctor, hospital, medicaid, afford, services, canada
35	2560	shapiro, ben, rubin, partisan, conservative, pakman, wing, harris, peterson, politcal, seder, sam, crowder, marriage, dave, interview, conservatives, rogan, podcast, daily
36	2650	marxism, marx, marxist, marxists, capitalism, cultural, revolution, communism, communist, capitalist, socialism, neo, ideology, proletariat, economic, production, blm, socialist, labor, communists
37	2681	peer, study, studies, review, research, reviewed, paper, papers, journals, published, meta, journal, scientific, science, reviewers, conclusions, publish, methodology, findings, results

38	1948	movie, movies, disney, characters, bond, film, character, hollywood, films, netflix, woke, marvel, star, idris, james, elba, cast, actor, actors, female
39	1887	bret, brett, weinstein, heather, eric, unity, evergreen, podcast, ballot, dawkins, dhs, sam, matt, plan, unity2020, evolutionary, audience, brets, grifter, ticket
40	2742	rubin, member, eric, weinstein, membership, ideologies, harris, conversations, rogan, sam, dave, topics, club, content, subreddit, peterson, shapiro, discussions, figures, movement
41	2742	conspiracy, theories, theorist, theorists, qanon, conspiracies, cia, hoax, 11, secret, conspiratorial, moon, cabal, mainstream, ufo, aliens, landing, believing, fake, trust
42	2355	statistics, coin, numbers, stats, sample, graph, statistical, 50, correlation, percentage, population, chart, estimates, 70, size, percent, survey, heads, statistic, average
43	1990	inflation, banks, currency, bitcoin, fed, loans, supply, debt, dollar, reserve, crypto, bank, printing, economy, gold, monetary, market, prices, financial, fiat
44	2558	rittenhouse, kyle, gun, shot, defense, police, shooting, officer, shoot, arrest, shooter, weapon, cops, rifle, officers, armed, rosenbaum, murder, knock, car
45	2173	nazis, nazi, hitler, nazism, germany, german, germans, jews, reich, socialists, party, national, socialist, jewish, neo, ideology, adolf, evil, socialism, aryan
46	2388	language, definitions, dictionary, english, meanings, usage, languages, semantics, define, dictionaries, linguistic, describe, vocabulary, changing, phrase, webster, defined, communicate, linguistics, labels
47	1590	israel, palestinians, israeli, palestinian, palestine, jews, arab, hamas, jewish, arabs, israelis, land, gaza, apartheid, peace, egypt, occupation, 1967, bank, conflict
48	3587	racist, nationalist, supremacist, nationalists, supremacy, racism, nationalism, supremacists, racists, race, obama, whites, racial, molyneux, stefan, president, whiteness, kkk, nazi, spencer
49	1933	mental, autism, illness, depression, disorder, diagnosis, autistic, health, trauma, therapy, disorders, psychology, diagnosed, mentally, psychiatry, therapist, schizophrenia, adhd, psychosis, personality
50	2058	rubin, dave, guests, rogan, seder, shapiro, interview, pakman, guest, joe, grifter, interviewer, interviews, alt, platform, audience, sam, peterson, criticism, patreon

51	1626	joe, rogan, spotify, podcast, guests, episodes, jre, alex, jones, joerogan, guest, bernie, mma, episode, cnn, interview, jack, comedian, fans, audience
52	2101	immigration, immigrants, border, borders, illegal, immigrant, migrants, replacement, asylum, migration, refugees, undocumented, ice, countries, mexico, wall, native, population, citizens, sanctuary
53	3301	burden, assertion, proven, provided, anecdotal, feelings, assertions, conclusion, presented, dismissed, convince, proves, factual, proving, counter, disprove, statements, providing, reasoning, logical
54	4098	echo, subs, chamber, subreddit, wing, leaning, conservative, chambers, subreddits, leftist, downvoted, leftists, content, upvotes, conservatives, mods, discussions, posting, users, space
55	2045	book, books, recommend, moloch, fiction, haidt, author, novel, audiobook, coddling, philosophy, chapter, recommended, maps, writing, series, jonathan, novels, righteous, recommendation
56	1378	sports, athletes, compete, trans, sport, female, men, mma, advantage, biological, male, testosterone, leagues, competing, team, players, soccer, competition, league, woman
57	1729	islam, muslims, muslim, islamic, quran, religion, christianity, christians, christian, sharia, cartoons, religious, religions, muhammad, mohammed, islamophobia, allah, western, west, europe
58	2628	morality, evil, suffering, immoral, morals, ethics, values, objective, ethical, morally, subjective, ought, nature, happiness, humans, pain, pleasure, altruism, harm, empathy
59	2167	videos, watched, youtube, watching, clip, minutes, audio, posted, content, title, bhi, minute, channel, hour, clips, documentary, tube, propaganda, speed, mins
60	1464	pronouns, pronoun, singular, gender, preferred, language, refer, plural, english, trans, male, gendered, female, sex, binary, sir, referred, woman, grammar, xe
61	1523	fascism, fascist, fascists, mussolini, eco, authoritarian, communism, socialism, ur, italy, nationalism, hitler, umberto, dictator, italian, ideology, nazi, nazis, totalitarian, authoritarianism
62	1761	marriage, gay, married, sex, parents, couples, sexual, marriages, religious, kids, marry, rights, bill, sexuality, child, lgbt, homosexuality, orientation, teachers, legal
63	1521	lab, leak, wuhan, virus, sars, cov, research, viruses, function, gain, bats, china, bat, fauci, origin, coronaviruses, coronavirus, virology, hypothesis, wiv

64	1612	libertarian, libertarians, libertarianism, liberty, freedom, party, authoritarian, liberal, liberalism, rand, market, private, nap, wing, taxes, conservatives, rights, economic, conservative, anarchism
65	1776	countries, eu, canada, europe, globalization, usa, european, americans, uk, canadian, brexit, nations, nation, united, canadians, western, norway, quebec, north, south
66	1684	harris, sam, podcast, klein, tds, islam, murray, weinstein, peterson, fan, jbp, charles, meditation, listened, seder, derangement, religion, episode, chomsky, clip
67	1922	socialism, socialist, capitalism, socialists, capitalist, production, ownership, communism, economy, democratic, economic, democracy, workers, countries, market, private, bernie, worker, welfare, owned
68	1688	tax, taxes, taxation, theft, income, pay, taxed, rich, wealth, paying, revenue, property, rate, paid, wealthy, sales, vat, spending, taxing, rates
69	1463	jews, jewish, semitism, semitic, jew, israel, antisemitism, judaism, semite, antisemitic, bolshevism, semites, christians, conspiracy, baddiel, ethnic, religion, religious, christian, holocaust
70	1562	equality, opportunity, equity, outcome, equal, meritocracy, merit, outcomes, fairness, opportunities, inequality, unequal, meritocratic, unfair, discrimination, achieve, education, born, inequalities, parents
71	3012	men, feminism, feminists, feminist, patriarchy, male, masculinity, toxic, female, gender, woman, equality, roles, sexual, sexist, sexism, masculine, rights, feminine, males
72	1134	eric, weinstein, portal, podcast, thiel, episode, sean, physics, peter, disc, brother, weinsteins, bret, sam, keefe, physicists, listening, geometric, lex, talks
73	1621	communism, communist, communists, capitalism, ussr, socialism, capitalist, soviet, utopia, marx, socialist, dictatorship, ideology, revolution, authoritarian, economic, production, stalin, china, countries
74	3350	virus, flu, covid, deaths, spread, lockdowns, pandemic, disease, immunity, lockdown, infected, measures, rate, hospitals, health, death, herd, infection, 19, die
75	1032	strike, charity, strikes, ban, removed, principle, applying, permanent, mischaracterizing, willfully, insults, charitable, brigading, trolling, third, debatelording, permaban, content, temp, attacks

76	1932	police, cops, officers, cop, policing, officer, enforcement, defund, crime, training, defunding, force, unions, reform, violent, accountability, arrest, departments, brutality, violence
77	1218	jones, alex, defamation, sandy, damages, hook, libel, trial, court, discovery, aj, jury, default, families, infowars, plaintiffs, sued, slander, lies, parents
78	986	drag, sexual, pageants, kids, queens, queen, child, sexualized, beauty, friendly, dress, dressing, kid, adult, performances, dancing, inherently, performance, burlesque, makeup
79	1837	objective, subjective, perception, truths, objectively, illusion, perceive, philosophy, existence, constructed, physical, concept, realism, shared, belief, universe, coherence, experiences, correspondence, perceptions
80	2697	crt, race, racism, racial, supremacy, racist, color, delgado, taught, whiteness, lens, teaching, stefancic, schools, whites, students, school, theorists, scholars, legal
81	1245	sam, seder, podcast, tds, samharris, episode, clip, dtg, rubin, dave, crowder, meditation, sams, listened, tribal, criticism, thinks, tribalism, ezra, bin
82	1845	gay, lgbt, sexual, homosexuality, orientation, queer, homosexual, sexuality, lgbtq, homophobic, straight, preference, sex, attracted, homophobia, heterosexual, born, gender, attraction, phobia
83	1047	books, book, library, seuss, libraries, publisher, publishing, publish, amazon, banning, publishers, dr, school, banned, burning, nea, mein, published, kampf, schools
84	1034	floyd, chauvin, neck, george, knee, death, died, autopsy, police, fentanyl, arrest, examiner, breathe, murder, killed, restraint, choke, cops, officer, overdose
85	1157	drugs, drug, alcohol, weed, marijuana, heroin, addiction, cocaine, legalization, meth, addicts, cannabis, fentanyl, substances, dealers, crack, illegal, possession, opium, prohibition
86	1212	joke, satire, comedy, sarcasm, jokes, funny, humor, sarcastic, comedians, parody, mocking, joking, laugh, humour, mockery, laughing, hilarious, laughed, poe, satirical
87	954	taliban, afghanistan, afghan, military, withdrawal, troops, kabul, biden, afghans, equipment, syria, leaving, soldiers, army, forces, civilians, botched, pakistan, afghani, evacuation
88	1068	id, voter, voting, ids, vote, laws, voters, fraud, ballot, polling, elections, mail, election, georgia, photo, requiring, hours, water, registration, license

89	1386	privilege, privileged, privileges, whites, advantages, skin, advantage, concept, color, race, racial, disadvantage, born, whiteness, wealth, racism, intersectionality, racist, blacks, guilt
90	3470	party, democrats, republicans, republican, dems, gop, democrat, democratic, parties, vote, voters, biden, policies, election, voting, obama, dnc, dem, platform, politicians
91	1459	rights, natural, property, constitution, liberty, locke, amendment, laws, freedom, violated, contract, inalienable, shall, healthcare, concept, bill, legal, water, protect, constitutional
92	1255	antifa, proud, boys, organization, violence, fascist, supremacists, fascists, blm, nazis, fascism, violent, supremacist, charlottesville, portland, prayer, patriot, organized, gavin, rally
93	1613	subreddit, subreddits, redditor, subs, users, redditors, karma, mods, downvote, red, upvotes, ruqqus, user, mod, site, downvoting, ant, admins, commenting, 4chan
94	889	web, dark, dialogue, weinstein, eric, refers, space, spanning, growing, peterson, harris, jordan, coined, held, mainstream, sam, variety, collection, subreddit, rogan
95	1778	democracy, democratic, republic, representative, vote, constitution, democracies, representatives, tyranny, voting, citizens, minority, elected, sortition, elections, governance, decisions, constitutional, oligarchy, consent
96	1798	deaths, covid, death, died, rate, numbers, excess, dying, mortality, die, 19, age, per, 000, million, cdc, hospital, vaccinated, unvaccinated, disease
97	1310	kids, parents, child, parenting, adults, age, adult, parent, kid, 18, adulthood, young, abuse, development, 25, maturity, teach, childhood, hitting, wife
98	1354	violence, violent, physical, speech, aggression, anger, harm, force, threats, assault, threat, aggressive, punch, abuse, language, incitement, silence, harmful, destructive, hurt
99	979	strawman, straw, steelman, steel, manning, strawmanning, steelmanning, motte, bailey, rephrase, fallacy, strawmen, fallacies, strawmanned, logical, whataboutism, weak, opponent, attacking, strongest
100	1062	actor, actors, engaging, accuse, hasan, accusing, intentions, intent, hominem, ad, respond, troll, operating, derail, responding, assumption, discussions, dialogue, accusations, lying
101	2005	capitalism, capitalist, market, profit, economic, markets, capital, production, labor, economy, capitalists, workers, property, wealth, private, socialism, communism, ownership, growth, worker

102	1300	identity, identities, idpol, race, racial, identify, individualism, intersectionality, focus, christian, labels, minorities, gender, party, color, divide, progressive, immutable, interests, ideology
103	1234	blm, movement, organization, alm, police, slogan, brutality, protests, blacks, mlk, marxist, violence, racism, racist, racial, supporting, protest, rioting, phrase, communities
104	707	yang, andrew, ubi, bernie, tulsi, candidate, gabbard, candidates, mcraven, vote, biden, warren, party, win, sanders, democratic, ticket, dem, endorsed, primary
105	763	attractive, beauty, makeup, beautiful, fat, sexual, attractiveness, wear, men, fashion, ideal, ugly, woman, body, swimsuit, workplace, magazine, magazines, standards, skinny
106	1241	solution, solutions, solve, fix, solved, solving, alternative, fixing, propose, solves, fixed, consequences, proposed, variables, solvable, viable, plan, soluble, redlining, problematic
107	1084	court, justices, supreme, judges, thomas, packing, courts, scotus, garland, judicial, justice, senate, constitution, judge, mcconnell, congress, republicans, democrats, recuse, filibuster
108	923	youtube, videos, content, crowder, channels, algorithm, channel, creators, maza, youtubers, platform, google, alt, ads, users, speech, algorithms, advertisers, recommendations, misinformation
109	1016	california, rural, texas, cities, blue, red, urban, areas, city, ca, tx, florida, californians, housing, austin, population, counties, vote, moving, area
110	851	consciousness, conscious, brain, agency, determinism, universe, choices, deterministic, physics, physical, illusion, decisions, predetermined, existence, decision, randomness, sam, quantum, brains, body
111	1015	rape, consent, sexual, assault, raped, accusations, victims, sex, woman, drunk, victim, men, rapes, accused, rapist, harassment, assaulted, rapists, accusation, sexually
112	738	loans, debt, loan, student, college, tuition, forgiveness, education, pay, bankruptcy, colleges, students, universities, degrees, cost, paid, paying, costs, school, financial
113	968	analogy, comparison, oranges, apples, compare, comparing, distinction, equivalence, differences, similarities, comparable, equivalent, comparisons, baseball, analogies, analogous, hole, compared, semantics, situations
114	989	genocide, camps, genocides, concentration, gulag, gulags, genocidal, ethnic, famine, prisoners, 000, armenian, camp, yugoslavia, atrocities, destruction, killed, lemkin, killing, nazi

115	891	mrna, gene, vaccines, protein, therapy, spike, vaccine, dna, cells, proteins, rna, immune, virus, malone, technology, cell, body, vector, nucleus, effects
116	1511	ubi, welfare, programs, income, yang, vat, tax, program, poverty, taxes, pay, benefits, jobs, spending, economy, net, month, assistance, 1000, unemployment
117	1117	historical, historians, taught, historian, zinn, events, teaching, books, slavery, learning, school, generations, ancient, crt, teach, repeat, ancestors, mistakes, hitler, 1984
118	1415	physics, universe, quantum, materialism, material, science, scientific, mechanics, simulation, theories, consciousness, model, popper, metaphysical, gravity, induction, models, metaphysics, epistemology, wave
119	622	tim, pool, rubin, crowder, wing, liberal, videos, dave, grifter, parkman, journalist, audience, joe, journalism, winger, poole, commentary, commentator, leaning, rogan
120	1387	party, parties, vote, voting, third, candidates, candidate, 3rd, votes, voters, election, ranked, duopoly, win, elections, electoral, primaries, unity, voted, republican
121	683	music, song, hop, hip, rap, songs, art, lyrics, cardi, wap, artists, musicians, pop, rapists, rock, artist, rappers, musical, rapist, musician
122	1048	automation, jobs, labor, automated, ubi, workers, ai, productivity, robots, unemployment, technology, machines, economy, hours, demand, worker, industries, production, workforce, labour
123	2216	wealth, inequality, rich, income, poverty, mobility, wealthy, middle, economic, gap, billionaires, economy, richer, capital, bottom, lower, tax, distribution, born, bezos
124	650	jp, jbp, fans, jps, lectures, religion, psychology, fan, believes, kb, advice, philosophy, atheist, criticism, daughter, listening, listened, subreddit, trunks, jung
125	1350	schools, school, education, teachers, kids, students, teacher, parents, teaching, funding, teach, student, curriculum, math, charter, schooling, private, learning, taught, district
126	999	postmodernism, modernism, postmodern, postmodernist, modernist, philosophy, marxism, postmodernists, neo, marxist, foucault, modernists, enlightenment, narratives, grand, frankfurt, ideology, objective, pluckrose, metanarratives
127	3976	conversations, debates, discussions, discourse, debating, beliefs, dialogue, discuss, disagreement, topics, productive, emotional, logical, engaging, positions, online, win, opposing, opponent, communication

128	589	google, search, results, duckduckgo, engine, engines, bing, algorithm, algorithms, searches, ddg, image, damore, images, knife, blake, duck, daisy, suggestions, apple
129	1083	evolution, evolutionary, species, biology, nature, humans, lobsters, selection, genes, environment, hierarchies, biological, genetic, natural, evolved, animals, gene, serotonin, nurture, apes
130	1382	iran, iraq, saudi, saddam, arabia, nuclear, iranian, military, invasion, weapons, yemen, syria, regime, saudis, oil, foreign, region, east, sanctions, isis
131	1119	fired, employer, employees, company, employee, fire, firing, employers, employment, workplace, speech, boss, companies, freedom, jobs, harassment, lose, hr, hire, religious
132	693	driving, drunk, seatbelt, car, trolley, drive, cars, seatbelts, seat, drivers, driver, belt, safety, accidents, speed, accident, wear, traffic, road, wearing
133	545	epstein, maxwell, jeffrey, pinker, dershowitz, suicide, pedophile, clinton, acosta, blackmail, island, powerful, ghislaine, trial, pedo, conspiracy, flight, jane, underage, girls
134	609	obesity, fat, obese, overweight, weight, healthy, health, exercise, food, diabetes, bmi, diet, eat, unhealthy, heart, disease, covid, eating, sugar, contagious
135	938	murder, penalty, death, killing, kill, punishment, prison, innocent, murderers, killed, execution, murderer, crime, executed, justice, guilty, murdered, executions, homicide, alive
136	842	dating, men, promiscuous, sex, apps, partners, incel, attractive, relationships, incels, sexual, relationship, casual, partner, date, promiscuity, tinder, males, woman, hypergamy
137	1256	court, courts, legal, supreme, judge, ruling, precedent, lawsuit, lawsuits, decision, judges, dismissed, scotus, lawyer, persky, lawyers, rulings, judicial, overturned, constitutional
138	1266	lie, lying, lies, lied, liar, liars, fauci, deception, dishonest, trust, caught, tells, oath, admitted, accusations, deer, quote, stories, believing, factual
139	1241	intellectuals, intellectualism, intellectually, intellectualdarkweb, intelligence, intelligent, wisdom, pseudo, intellect, smart, subreddit, thinkers, ignorance, awareness, dishonest, smarter, academic, educated, discourse, philosophy
140	1092	men, female, gender, stem, male, gap, fields, sexism, career, differences, workforce, jobs, roles, woman, pay, 50, discrimination, girls, engineering, equality

141	653	tribalism, tribe, tribal, tribes, tribalistic, identity, oxytocin, humans, tribalist, gun, collective, ethnic, nature, instincts, beliefs, identities, communities, indian, subreddit, blue
142	551	tucker, carlson, oliver, fox, maddow, john, viewers, greenwald, immigration, 2017, replacement, nyt, rachel, racist, conservative, immigrants, nationalist, whistles, stewart, dirtier
143	703	civil, military, armed, unrest, conflict, violence, army, wars, tanks, militia, warfare, militias, factions, uprising, weapons, fighting, domestic, guerrilla, guns, antifa
144	684	anarchist, anarchism, anarchy, anarchists, bookchin, anarcho, communalism, hierarchies, communalist, hierarchy, communes, institutions, libertarian, assemblies, ecological, capitalism, statist, zapatistas, forms, commune
145	949	scared, afraid, mongering, paranoid, fears, scary, scare, paranoia, panic, threat, terrified, fearmongering, worry, terrifying, worried, fearful, trust, covid, safe, danger
146	723	sex, sexual, minors, prostitution, consent, pedophilia, porn, child, stigma, pedophiles, trafficking, boss, abuse, pedophile, rape, adults, attracted, consenting, minor, workplace
147	562	pakman, david, progressive, rubin, pool, seder, shapiro, progressives, kulinski, tim, dore, fans, crowder, partisan, interview, india, content, youtube, videos, kyle
148	485	meat, vegan, diet, eat, vegans, veganism, eating, animal, food, vegetarian, animals, foods, diets, cattle, beef, plant, nutrients, vegetables, protein, cows
149	1165	cultures, cultural, appropriation, pride, values, proud, european, western, differences, culturally, nations, americans, immigrants, norms, ethnic, music, shared, asian, customs, traditions
150	832	troll, trolling, trolls, bye, trolled, blocked, feed, actor, mods, posting, engaging, waste, provoke, user, baiting, bait, malice, responding, deliberately, insults
151	531	ben, neil, shapiro, interview, interviewer, partisan, andrew, marriage, politcal, conservative, bbc, daily, rush, wing, immoral, gay, dave, style, rubin, podcast
152	519	flag, confederate, symbol, flags, fly, confederacy, burning, symbols, flying, swastika, southerners, represents, slavery, southern, south, racist, rebel, pride, burn, nazi
153	475	art, ai, artist, artists, artistic, banana, creative, tool, arts, skill, digital, taped, canvas, technical, artwork, generated, music, creativity, blank, paintings

154	1165	nuance, lmao, que, thee, upvote, asterick, unironically, es, de, nuanced, autocorrect, spelled, hypocrisy, acronyms, en, jab, mone, autistic, tize, da
155	773	property, land, ownership, private, rights, labor, owner, violence, natural, owns, defend, theft, contract, owned, rent, taxes, consent, force, enforce, possession
156	1094	laws, legal, illegal, legislation, unconstitutional, enforce, enforced, legally, morality, constitution, court, apply, courts, statute, legality, centralized, crime, applied, jacobson, judges
157	498	laptop, hunter, emails, biden, giuliani, repair, fbi, rudy, contents, confirmed, verified, hacked, computer, disinformation, drive, joe, fake, email, shop, authentic
158	519	animals, animal, pets, meat, eat, humans, pet, eating, sentient, suffering, mice, dogs, dog, killing, species, consent, pleasure, food, kill, torture
159	586	bathroom, trans, bathrooms, prisons, prison, cis, woman, men, rooms, restroom, spaces, male, female, restrooms, locker, transwomen, sex, males, transgender, gender
160	1466	trial, guilty, jury, verdict, convicted, charges, murder, innocent, court, chauvin, charged, crime, prosecution, justice, criminal, defense, judge, convict, accused, charge
161	929	freedom, liberty, freedoms, liberties, safety, rights, security, restrictions, tyranny, laws, constitution, autonomy, property, temporary, happiness, responsibility, protect, choices, limits, consequences
162	444	lindsay, james, pluckrose, helen, twitter, woke, lindsey, crt, discourses, boghossian, mlh, book, lindsays, theories, podcast, marc, phd, tweet, cynical, hegel
163	670	prison, prisons, prisoners, criminals, crime, jail, bail, crimes, inmates, recidivism, private, rehabilitation, offenders, criminal, justice, violent, incarceration, punishment, rehabilitated, profit
164	540	hunter, biden, joe, son, burisma, kushner, corruption, corrupt, laptop, board, shokin, company, jared, father, president, ivanka, prosecutor, dad, emails, investigating
165	878	bias, biased, biases, confirmation, implicit, unbiased, misinformation, unconscious, neutral, study, objective, cognitive, articles, skepticism, beliefs, unreliable, studies, worldview, algorithm, accuracy
166	1043	tds, derangement, biden, dementia, syndrome, president, debates, decline, cognitive, supporters, mental, joe, stutter, obama, donald, aphasia, presidential, hours, brain, presidency

167	746	lockdowns, lockdown, sweden, lock, measures, restrictions, deaths, covid, locked, pandemic, masks, mandates, downs, virus, economy, countries, policies, locking, australia, compliance
168	779	vax, vaxxers, vaxx, vaxxer, antivax, vaccination, antivaxxers, vaccinated, vaush, vaxxed, pro, covid, antivaxxer, mandate, mandates, vaccinations, medical, antivaxx, hoax, unvaccinated
169	1730	articles, headline, title, author, paper, com, posted, quote, linked, misinformation, published, headlines, misleading, shockley, gunn, editor, publication, mikhaila, paragraph, content
170	887	wage, wages, minimum, labor, workers, pay, employees, jobs, paid, worker, price, businesses, market, increase, profit, prices, inflation, productivity, employee, company
171	578	ngo, andy, antifa, journalist, portland, journalists, violence, attacked, patriot, reporting, prayer, proud, quillette, fa, boys, footage, assaulted, assault, victim, wing
172	1185	wikipedia, citation, citations, links, cite, references, citing, credible, research, google, quote, cited, provided, reliable, refute, academic, linked, reference, linking, page
173	625	alt, lite, spencer, neo, nazi, movement, groypers, wing, milo, gateway, righters, richard, nazis, label, altruism, conservative, sargon, nationalism, nationalists, supremacists
174	877	economics, economists, economic, austrian, economist, economy, sowell, mises, gdp, market, keynesian, trade, keynes, econ, book, growth, empirical, stock, price, trickle
175	606	holocaust, jews, jewish, camps, nazi, bolshevik, germany, genocide, german, denier, nazis, jew, denial, bolsheviks, million, bolshevism, hitler, concentration, germans, historical
176	802	propaganda, misinformation, disinformation, lies, russian, agenda, spreading, fake, newspeak, spread, brainwashing, manipulation, masses, misleading, brainwashed, narratives, manipulated, ideology, techniques, divisive
177	770	victim, victimhood, shaming, victims, honor, abuse, shame, blaming, responsibility, victimized, oppressed, victimization, guilt, trauma, hurt, complicit, empathy, harmed, honour, bully
178	858	delta, variants, variant, vaccine, virus, vaccinated, vaccines, mutations, infection, immunity, antibodies, mutation, immune, unvaccinated, vaccination, spike, resistant, infected, sars, protein
179	1479	vote, voting, electoral, votes, ranked, ec, college, voters, elections, electors, representation, democracy, representatives, candidates, senate, election, candidate, popular, voter, representative

180	847	ford, kavanaugh, investigation, testimony, allegations, fbi, committee, hearings, oath, court, senate, witnesses, trial, hearing, guilty, democrats, lying, accusation, assault, perjury
181	454	cult, cults, scientology, leader, cultish, religion, member, culty, rand, cultist, atheism, leaders, followers, religions, belief, autistic, mormons, impending, beliefs, ideology
182	721	generation, gen, boomers, millennials, generations, young, younger, older, age, youth, millennial, boomer, parents, kids, generational, genx, olds, lived, progress, millenials
183	630	coup, attempted, overthrow, election, coups, etat, capitol, president, military, insurrection, legal, plan, democracy, attempting, regime, elected, votes, revolution, officials, violent
184	702	housing, rent, house, home, property, houses, homes, zoning, prices, market, income, landlords, afford, mortgage, buy, renting, rents, rental, price, estate
185	1040	obama, drone, president, strikes, iran, appeasement, peace, isis, military, syria, biden, wars, administration, foreign, yemen, iraq, korea, kurds, civilian, bush
186	438	australia, nz, australian, quarantine, australians, zealand, melbourne, lockdown, lockdowns, victoria, maori, camps, aussie, aussies, travelers, covid, vic, hotel, aus, restrictions
187	586	western, west, civilization, values, roman, rome, huntington, empire, eastern, east, europe, enlightenment, romans, civilizational, european, cultures, countries, greek, greeks, civilizations
188	495	oil, prices, gas, supply, energy, price, production, pipeline, biden, drilling, demand, fuel, keystone, russia, global, barrels, drill, inflation, fossil, crude
189	631	pronouns, 16, misgendering, harassment, bill, compelled, pronoun, speech, canadian, peterson, discrimination, gender, court, tribunal, preferred, jail, ohrc, canada, c16, cossman
190	712	stalin, communism, soviet, communists, communist, nazis, nazism, nazi, ussr, hitler, gulags, million, killed, famine, churchill, germany, mao, union, soviets, millions
191	493	murray, douglas, crowds, madness, charles, book, interview, europe, interviews, books, strange, immigration, harris, insinuations, hitchens, identity, ayaan, csc, bell, sam
192	347	flat, earth, earthers, round, earther, conspiracy, spherical, burden, malone, sun, model, john, theorists, videos, consensus, youtube, curvature, delusion, shape, theories

193	561	ai, agi, intelligence, machine, brain, machines, artificial, humans, computer, neural, algorithm, robot, software, learning, hardware, consciousness, computation, turing, algorithms, programming
194	411	delta, variant, vaccinated, variants, unvaccinated, viral, mutations, spread, omicron, strain, virus, mutation, alpha, load, breakthrough, contagious, infectious, infected, loads, viruses
195	1053	federal, constitution, local, rights, laws, amendment, constitutional, union, governments, powers, legislature, commerce, federalism, united, national, jurisdiction, court, congress, sovereign, interstate
196	1303	violence, wing, terrorism, extremists, violent, attacks, antifa, csis, threat, extremism, terrorist, leftist, shooting, killed, mass, wingers, shooter, domestic, dangers, extremist
197	539	colonialism, indigenous, native, land, colonization, british, colonial, natives, europeans, european, colonies, colonized, tribes, colonists, africa, conquest, americans, plymouth, indians, empire
198	1213	bill, senate, pass, bills, republicans, legislation, congress, stimulus, filibuster, dems, passed, senators, democrats, bipartisan, omnibus, relief, gop, vote, mcconnell, republican
199	444	math, mathematics, mathematical, mathematicians, numbers, triangle, symbols, arithmetic, maths, language, abstract, objects, subjective, circle, geometry, degrees, axioms, concepts, universe, beauty
200	883	vaccinated, vaccination, vax, vaccinations, unvaccinated, vaxxed, covid, mandate, vaccinate, mandates, vaers, vaccinating, testing, status, mandatory, mandated, require, vaxx, unvaxxed, decision
201	716	terrorism, terrorists, terrorist, domestic, stochastic, terror, violence, threat, blm, violent, civilians, attacks, acts, capitol, antifa, threats, isis, organization, bombings, 11
202	389	venezuela, cuba, maduro, chavez, cuban, castro, venezuelan, venezuelans, chile, socialist, latin, regime, sanctions, colombia, cubans, guaido, countries, oil, opposition, haiti
203	1002	corporations, companies, corporate, tech, company, corporation, woke, employees, profit, businesses, valley, amazon, market, silicon, capitalism, koch, wing, leftist, thiel, liberals
204	422	bot, amp, summon, bots, amputatorbot, canonical, haikusbot, load, fakespot, privacy, faster, reviews, web, opt, optout, detect, page, concerns, controversial, shared
205	339	diet, nutrition, sugar, food, fat, dietary, keto, eat, eating, health, processed, diets, carnivore, nutritional, weight, calories, meat, ede, disease, autism

206	574	ct, critique, theorists, frankfurt, structures, crt, theories, postmodernism, horkheimer, lindsay, philosophy, marxism, criticism, science, ideology, pedagogy, critiques, method, critically, book
207	765	offended, offensive, offense, insult, offend, insults, triggered, joke, insulted, upset, language, insulting, offensiveness, jokes, offence, slurs, outrage, comedian, sensitive, reaction
208	547	family, nuclear, families, parent, parents, disrupt, father, mothers, marriage, fathers, divorce, extended, kids, child, mother, household, married, structure, monogamy, raising
209	611	privacy, surveillance, encryption, tech, digital, nsa, private, phone, companies, snowden, technology, access, tracking, app, patriot, security, transparency, google, spying, backdoor
210	862	authoritarian, authoritarianism, authoritarians, authority, freedom, totalitarian, dictator, governments, totalitarianism, junta, policies, tendencies, norm, liberty, measures, democracy, opposition, tyranny, force, libertarian
211	382	japan, japanese, globalism, imperialism, genocidal, western, immigration, empire, abe, imperialists, imperial, asian, nations, china, genocide, countries, globalists, imperialist, empires, pearl
212	420	ads, ad, gillette, advertising, marketing, commercial, advertisement, advertisers, product, masculinity, toxic, behaviour, men, brand, content, gillete, products, cola, advert, coke
213	574	rationality, irrational, rationally, irrationality, rationalists, rationalism, rationalist, emotions, logical, rationalwiki, reasoning, emotional, rationalize, yudkowsky, emotion, eliezer, intuition, praxeology, rationale, decision
214	304	suicide, suicides, suicidal, men, suicidality, anorexia, males, depression, male, rates, rate, commit, questionnaire, feelings, mental, deaths, kill, sommers, attempts, die
215	550	covid, 19, polio, pandemic, contagious, covidiot, catching, booster, denier, mild, covidian, unvaccinated, deadly, lockdowns, covidians, psychosis, spread, illogical, tired, dying
216	489	unions, union, workers, labor, strike, company, worker, employees, bargaining, striking, jobs, unionized, companies, employer, teachers, contract, employers, wages, pay, sector
217	785	removed, contribution, mods, deleted, content, removal, violation, moderators, deemed, user, users, attacks, quality, adding, mod, standards, substance, contributing, removing, hominem
218	329	klein, ezra, vox, harris, sam, haidt, podcast, sullivan, interview, ethan, bigoteer, murray, identity, listening, charles, dishonest, conversations, ben, shapiro, justice

219	919	nazis, supremacists, condemn, neo, wallace, supremacy, rally, proud, boys, condemned, antifa, stand, charlottesville, nazi, march, statue, marching, violence, nationalists, denounce
220	361	discord, server, join, meetup, invite, meetups, chat, dm, angeles, los, club, conversations, pm, topics, channel, portal, discussions, podcast, hour, hi
221	2024	movement, movements, activism, activists, activist, blm, ideology, radical, protests, progressive, protest, revolution, woke, collective, leaders, institutions, goals, progress, oppression, status
222	298	statues, statue, confederate, monuments, jefferson, columbus, erected, washington, slavery, monument, symbol, museum, toppling, slaves, celebrate, slave, historical, tear, celebrating, removing
223	474	crt, cr, taught, proponents, schools, rufo, delgado, academic, cringe, bell, advocates, wikipedia, school, teaching, lens, define, elementary, grows, diangelo, scholars
224	744	immunity, natural, immune, antibodies, infection, cells, antibody, covid, herd, memory, vaccine, vaccinated, disease, vaccination, infected, virus, protection, pathogen, sars, vaccines
225	1330	democrats, party, republicans, voters, democratic, blacks, mlk, republican, civil, whites, racist, democrat, vote, southern, king, racial, negro, rights, race, americans
226	857	hierarchy, utopia, bookchin, hierarchies, utopian, ecology, hierarchical, nature, societies, ecological, domination, systems, forms, humans, ideal, emergence, development, vision, resources, humanity
227	429	fraud, court, courts, fraudulent, lawsuits, giuliani, widespread, dominion, baseless, audits, allegations, alleging, testimony, audit, customer, proven, sourced, corruption, irregularities, fake
228	238	beep, boop, robot, bot, copy, info, books, republic, manifesto, 1984, frankenstein, das, koran, kapital, communist, leviathan, heidi, punishment, macbeth, bop
229	388	homeless, homelessness, housing, cities, shelter, mental, city, addiction, streets, drug, addicts, shelters, drugs, poverty, rent, la, sf, san, food, health
230	422	ted, unsubstantiated, unvaccinated, tweeted, misinterpreted, tedious, obligated, retweeted, convoluted, kaczynski, cruz, uninterested, substantiated, solicited, envy, indoctrinated, farted, advice, unsolicited, unsophisticated
231	294	jordan, jordanpeterson, jbp, interview, jordans, atheist, jp, hall, peterson, milo, lectures, talks, futurist, message, podcast, metaphysical, sam, jordanbpeterson, helped, vibe

232	382	dave, daverubin, guests, interview, joe, chappelle, rogan, guest, rubin, sam, interviews, seder, interviewer, chapelle, criticism, ana, ruben, comedy, molyneux, ben
233	628	fertility, kids, population, birth, rates, child, growth, babies, birthrates, countries, replacement, eugenics, family, parental, decline, reproduce, rate, birthrate, overpopulation, families
234	404	bret, vaccines, ivermectin, vaccine, brett, covid, heather, roulette, ivm, sam, podcast, weinstein, safe, vaccinated, effective, risks, vax, study, kory, russian
235	1486	technology, progress, technological, humanity, tech, civilization, technologies, humans, global, century, innovation, species, growth, wisdom, collapse, planet, optimistic, systems, energy, resources
236	1491	vaccinated, virus, infected, unvaccinated, infection, covid, spread, viral, vaccination, symptoms, transmission, asymptomatic, load, viruses, pcr, infections, infectious, immunity, disease, sick
237	342	hat, nfl, maga, kneeling, anthem, wearing, kaepernick, hats, wear, shirt, nike, hijab, colin, players, knee, symbol, flag, kneel, football, protest
238	676	trans, rowling, jk, transphobic, woman, gender, transgender, sex, biological, book, menstruate, female, transphobia, identity, shrier, activists, transition, men, lgbt, walsh
239	792	racist, native, racism, cherokee, fragility, warren, race, woman, color, book, ancestry, skin, diangelo, whites, racial, blackface, indian, colour, racially, whiteness
240	931	oppression, minority, minorities, oppressed, oppressive, injustice, marginalized, tyranny, rights, whites, dominant, privilege, ethnic, discrimination, race, identity, dominance, oppress, equal, oppressor
241	773	lobbying, politicians, lobbyists, donations, campaign, bribery, corruption, politician, donate, pacs, corporations, campaigns, donors, pac, congress, elected, lobbyist, candidates, bribe, candidate
242	671	hospitals, hospital, beds, icu, patients, hospitalizations, covid, nurses, hospitalization, unvaccinated, capacity, vaccinated, overwhelmed, hospitalized, staff, bed, numbers, medical, healthcare, surgeries
243	297	france, french, muslims, macron, muslim, islam, islamic, revolution, condemn, paris, republic, attacks, islamist, secular, beheading, christchurch, king, hebdo, charlie, terrorism
244	514	jp, friendship, sil, wife, jerk, boundaries, relationship, blocked, uncomfortable, exfriend, trans, exfriended, subreddit, emotional, sister, friendships, advice, girlfriend, nazi, offended

245	671	cultural, wars, conservative, counterculture, fighting, appropriation, outrage, conservatives, divide, crt, cancel, conflict, twitter, topics, counter, conquer, win, cold, mainstream, online
246	1076	school, students, teachers, schools, teacher, crt, teaching, taught, bill, teach, curriculum, race, parents, kids, racist, student, discomfort, board, education, classroom
247	406	nationalism, patriotism, nation, nationalist, national, ethnic, proud, patriot, exceptionalism, nationalists, identity, patriotic, european, civic, patriots, pride, nations, nationalistic, values, diversity
248	494	military, army, combat, soldiers, draft, service, conscription, marine, veterans, marines, join, vets, wars, troops, recruits, soldier, men, corps, enlist, iraq
249	516	justice, sjw, sjws, warrior, movement, ideology, warriors, injustice, progressivism, solidarity, progressive, equality, rights, civil, lindsay, values, racism, movements, discrimination, pluckrose
250	273	benzos, benzo, addiction, prescribed, addicted, withdrawal, anxiety, taper, drugs, peterson, detox, addict, coma, benzodiazepines, daughter, turkey, wife, mikhaila, drug, rehab
251	341	korea, north, vietnam, nk, korean, south, marines, naval, china, koreans, troops, yeonmi, defectors, park, land, forces, japan, vietnamese, khmer, panama
252	197	boop, beep, robot, bot, copy, info, bible, books, quran, comics, robots, intend, promote, leaving, , , , , ,
253	300	ad, hominem, hom, hominems, homs, fallacy, insult, character, attacks, fallacies, attacking, maher, insults, fallacious, adl, mod, logical, cursewords, interlocutor, insulting
254	460	college, degrees, education, jobs, school, graduates, graduate, university, majors, certifications, engineering, bachelor, debt, skills, educated, colleges, career, students, require, trades
